# Short-Term Dendritic Dynamics of Neonatal Cortical Neurons Revealed by *In Vivo* Imaging with Improved Spatiotemporal Resolution

**DOI:** 10.1523/ENEURO.0142-23.2023

**Published:** 2023-11-07

**Authors:** Luwei Wang, Shingo Nakazawa, Wenshu Luo, Takuya Sato, Hidenobu Mizuno, Takuji Iwasato

**Affiliations:** 1 Laboratory of Mammalian Neural Circuits, National Institute of Genetics, Mishima 411-8540, Japan; 2 Graduate Institute for Advanced Studies, SOKENDAI, Mishima 411-8540, Japan; 3 International Research Center for Medical Sciences, Kumamoto University, Kumamoto 860-0811, Japan

**Keywords:** barrel cortex, layer 4, mouse, neonatal brain, neuronal circuit refinement, time-lapse imaging

## Abstract

Individual neurons in sensory cortices exhibit specific receptive fields based on their dendritic patterns. These dendritic morphologies are established and refined during the neonatal period through activity-dependent plasticity. This process can be visualized using two-photon *in vivo* time-lapse imaging, but sufficient spatiotemporal resolution is essential. We previously examined dendritic patterning from spiny stellate (SS) neurons, the major type of layer 4 (L4) neurons, in the mouse primary somatosensory cortex (barrel cortex), where mature dendrites display a strong orientation bias toward the barrel center. Longitudinal imaging at 8 h intervals revealed the long-term dynamics by which SS neurons acquire this unique dendritic pattern. However, the spatiotemporal resolution was insufficient to detect the more rapid changes in SS neuron dendrite morphology during the critical neonatal period. In the current study, we imaged neonatal L4 neurons hourly for 8 h and improved the spatial resolution by uniform cell surface labeling. The improved spatiotemporal resolution allowed detection of precise changes in dendrite morphology and revealed aspects of short-term dendritic dynamics unique to the neonatal period. Basal dendrites of barrel cortex L4 neurons were highly dynamic. In particular, both barrel-inner and barrel-outer dendrites (trees and branches) emerged/elongated and disappeared/retracted at similarly high frequencies, suggesting that SS neurons acquire biased dendrite patterns through rapid trial-and-error emergence, elongation, elimination, and retraction of dendritic trees and branches. We also found correlations between morphology and behavior (elongation/retraction) of dendritic tips. Thus, the current study revealed short-term dynamics and related features of cortical neuron dendrites during refinement.

## Significance Statement

The formation of proper dendritic patterns during early postnatal development is essential for normal neuronal circuit function in adulthood. To elucidate the mechanisms responsible for this refinement, *in vivo* imaging with high spatiotemporal resolution is useful. Our previous long-term *in vivo* imaging studies have clarified aspects of dendritic refinement mechanism; however, because of the long intervals (8 h) between image acquisitions, rapid changes in dendritic morphology were missed. Here hourly *in vivo* time-lapse imaging of neonatal mouse barrel cortex over 8 h revealed the rapid changes in the dendrite morphology of layer 4 neurons, thereby providing a more comprehensive record of dendritic refinement during postnatal development for mechanistic analysis.

## Introduction

Complex yet precise connectivity of neurons underlies higher brain functions in mammals. The specificity of neuronal connections depends largely on dendritic patterns as they determine the inputs received by individual neurons. Mature spiny stellate (SS) neurons in the mouse primary somatosensory cortex (barrel cortex) layer 4 (L4) show highly biased basal dendritic patterns oriented toward the barrel center, where thalamocortical axons (TCAs) transmit single-whisker inputs. This unique asymmetric basal dendritic pattern, which underlies the precise one-to-one functional relationship between whiskers and barrels, is formed during neonatal stages in a TCA input-dependent manner (Datwani et al., 2002; Espinosa et al., 2009; Mizuno et al., 2014). Thus, barrel cortex SS neuron basal dendrites are an ideal model for studying the mechanisms of dendritic refinement.

To reveal the mechanisms of dendritic refinement, it is critical to elucidate the dynamic features of dendrites during early postnatal development. Two-photon microscopy has been used extensively for imaging of spine dynamics *in vivo* during late postnatal development and adulthood (Lendvai et al., 2000; Holtmaat and Svoboda, 2009; Yang et al., 2009). Some studies have also addressed axon dynamics *in vivo* (Ruthazer et al., 2003; Portera-Cailliau et al., 2005; Carrillo et al., 2013). On the other hand, understanding dendrite dynamics is largely delayed (Mizuno et al., 2014; Nakazawa et al., 2018), which is partly because visualization of dendrite morphology *in vivo* is difficult in the neonatal brain (Young et al., 2008; Espinosa et al., 2009; Ako et al., 2011; Luo et al., 2016). We previously achieved that by developing the Supernova system, a versatile sparse cell-labeling method (Mizuno et al., 2014; Luo et al., 2016), and successfully performed two-photon *in vivo* imaging of individual L4 neurons in the neonatal barrel cortex at four time points within 18 h between postnatal day 5 (P5) and P6 (Mizuno et al., 2014). This study analyzed morphologic changes in barrel cortex L4 neuron basal dendrites in the neonatal cortex and detected dendritic motility *in vivo* for the first time. Subsequently, we achieved longitudinal *in vivo* imaging of morphologic changes in basal dendrites from P3 to P6 (Nakazawa et al., 2018).

Although the aforementioned study revealed the long-term dynamics that generate orientation bias, the spatiotemporal resolution was not sufficient to capture short-term dendritic dynamics during neonatal dendritic refinement. In that study, to cover the whole refinement process between P3 and P6, we set acquisition intervals of 8 or 24 h. However, the changes in dendrite morphology over 8 h are much larger than expected (Nakazawa et al., 2018), which hindered the detection of rapid changes over shorter periods. For example, if a dendritic branch is eliminated and a new branch emerges at a similar position within 8 h, it could be mistakenly recognized as the same. Thus, greater temporal resolution is required to reduce such potential errors.

To characterize the short-term dynamics of SS neuron dendrites more precisely, we here conducted hourly imaging for 8 h. We also improved the spatial resolution by labeling cell surfaces with a membrane-binding fluorescent protein, which was helpful to visualize more precise dendrite morphology. In the current study, we describe the short-term dynamics of cortical neuron dendrites during the critical period of dendritic refinement. Also, we report that the elongation (E) and retraction (R) of dendrites were positively correlated with dendrite tip (T) thickness.

## Materials and Methods

### Animals

All experiments were performed according to guidelines for animal experimentation of National Institute of Genetics (NIG) and were approved by the animal experimentation committee of NIG. TCA-green fluorescent protein (GFP) transgenic (Tg) mice (Mizuno et al., 2014) were backcrossed from C57BL/6J to ICR mice more than four times and were intercrossed to obtain TCA-GFP Tg homozygous male mice. Homozygosity of the TCA-GFP Tg mice was determined by quantitative PCR genotyping using the primers KS89 (TTCTCGTTGGGGTCTTTGCT) and KS90 (ACTTCTTCAAGTCCGCCATG). To obtain pups, ICR female mice were mated with the TCA-GFP Tg homozygous male mice. The day at which the vaginal plug was detected (between 10:00 A.M. and 11:00 A.M.) was designated as embryonic day 0 (E0), and E19 was defined as P0.

### Plasmids

The following Flpe/FRT-based Supernova vectors were used for sparse labeling: pK036 (TRE-Flpe-WPRE; Luo et al., 2016); pK037 [CAG-FRT-STOP-FRT-turbo red fluorescent protein (turboRFP)-ires-tTA-WPRE; Luo et al., 2016]; and pK300 (CAG-FRT-STOP-FRT-GAP43tagRFP-ires-tTA-WPRE). pK302 [CAG-blue fluorescent protein (BFP)-WPRE; Nakazawa et al., 2020] was used for dense labeling. The plasmid DNA was purified by a Midi Prep Kit (MACHEREY-NAGEL). For the generation of pK300, the GAP43tagRFP sequence was obtained from pK190 (CAG-loxP-STOP-loxP-GAP43tagRFP-ires-tTA-WPRE) vector by SalI/EcoRV restriction digestion and inserted into SalI/EcoRV sites of pK068 (CAG-FRT-STOP-FRT-EGFP-ires-tTA-WPRE vector; Luo et al., 2016). For the generation of pK190, GAP43 sequence (Chiaramello et al., 1996) and tagRFP sequence from pTagRFP-N vector (Kozak, 1987; Haas et al., 1996) were fused and PCR amplified using the primers HM67 (CTAGTGTCGACATGCTGTGCTGTATGAGAAGAACCAAACAGGTTGAAAAGAATGATGAGGACCAAAAGATCGAG) and HM75 (CCGATATCTTCAATTAAGTTTGTGCCCCAGTTTGCTAGG), and then inserted into the SalI/EcoRV sites of pK038 (CAG-loxP-STOP-loxP-EGFP-ires-tTA-WPRE) vector (Luo et al., 2016).

### *In utero* electroporation

*In utero* electroporation (IUE) was performed on TCA-GFP Tg heterozygous mice at E14 between 10:00 A.M. and 12:00 P.M. to label L4 neurons in the barrel cortex. The pregnant mothers were anesthetized via intraperitoneal injection of triple anesthesia (11 μg/g body weight). The triple anesthesia contains medetomidine hydrochloride (0.75 μg/g), midazolam (4 μg/g), and butorphanol tartrate (5 μg/g). A total of 0.5 μl of DNA solution (mixed with 5% methylene blue) was injected into the right lateral ventricle of embryos via a pulled glass capillary (Drummond), and square electric pulses (40 V; 50 ms) were delivered 5 times at 1 Hz by a CUY21EDIT electroporator (NepaGene). The electric pulses were given three times/embryo, and the current was 70–100 mA/electric pulse. After the IUE, pregnant mothers were injected with an antagonist (11 μg/g body weight), which contained atipamezole hydrochloride (0.75 μg/g), to wake them up from anesthesia and were kept on a 37°C heater until they recovered from anesthesia.

For Supernova-cytosolic RFP (cRFP) labeling, a DNA solution containing pK036 (20 ng/μl) and pK037 (1 μg/μl) was used. For Supernova-membrane-bound RFP (mRFP) labeling, a DNA solution containing pK036 (20 or 40 ng/μl), pK300 (1 μg/μl), and pK302 (200 ng/μl) was used.

### *In vivo* imaging with two-photon microscopy

For *in vivo* imaging of L4 neurons in the barrel cortex, TCA-GFP Tg heterozygous pups at P4 were used, in which L4 neurons were labeled by IUE-based BFP and Supernova-mRFP. Mice in which L4 neurons in the barrel cortex were labeled appropriately were identified by BFP fluorescence over the skull after removing the skin from the corresponding areas. Then, the craniotomy was performed on the right half of the head while the pups were under isoflurane anesthesia (3%), as described previously (Mizuno et al., 2014; Nakazawa et al., 2018). Briefly, the skull above the IUE-labeled area in the barrel cortex was carefully removed by a razor blade with the dura remaining intact, and a small round cover glass (diameter, 2.5 mm; Matsunami) was placed to cover the open skull area. Gelfoam (Pfizer) was used to stop the occasional minor bleeding and cortex buffer (Holtmaat et al., 2009; 125 mm NaCl, 5 mm KCl, 10 mm glucose, 10 mm HEPES, 2 mm CaCl_2_, and 2 mm MgSO_4_; pH 7.4) was applied to the skull-removed area to keep the brain moist during surgery. A customized titanium bar (weight, ∼30 mg; Nakazawa et al., 2018) was attached to the area adjacent to the cranial window. No apparent surgery-induced discomfort was observed. After 1 h of recovery on a 37°C heater with littermates, pups with cranial windows were used for *in vivo* imaging. Pups were anesthetized with isoflurane (0.9−1.4%) and head fixed to the microscope stage using the titanium bar during *in vivo* imaging. A heating pad was used to keep the pups warm. Images were acquired using a two-photon microscope (model LSM 7MP, Zeiss) with a W Plan-Apochromat 20×/1.0 differential interference contrast objective lens (Zeiss) and an LSM BiG detector (Zeiss). A HighQ-2 laser (Spectra-Physics) at 1045 nm was used in all experiments. GFP and RFP were simultaneously excited, and the emitted fluorescence was filtered (GFP, 500−550 nm; RFP, 575−620 nm). *In vivo* imaging was performed as follows: hour 0 (h0), h1, h2, h3, h4, h5, h6, h7, and h8 sessions started around; 1:00, 2:00, 3:00, 4:00, 5:00, 6:00, 7:00, 8:00, and 9:00 P.M. at P4, respectively. Each imaging session took ∼20 min. Between two imaging sessions, pups were returned to their littermates and kept on a 37°C heater. The pups usually start to respond to touch from littermates and move a few minutes after the end of the imaging session.

### Histology and confocal microscopy

After *in vivo* imaging, brain samples were obtained and fixed with ice-cold 4% paraformaldehyde in 0.1 m phosphate buffer (PB). The brain samples were kept at 4°C overnight and protected from light. For tangential sectioning, the right hemispheres were flattened and transferred to 30% sucrose in 0.1 m PB and kept overnight at 4°C. Tangential sections (thickness, 100 μm) of the flattened cortices were made using a freezing microtome (model ROM-380, Yamato).

Three-dimensional (3D) fluorescence images were acquired using a confocal microscope (model TCS SP5, Leica). Images of the barrel map and labeled neurons were taken with a 10×/0.4 CS2 objective lens with 2× digital zoom at a step size of ∼2.5 μm, and the total thickness was ≤100 μm. The 20× images were taken with 1024 × 1024 pixels. Images of fine structures of dendrites were taken with a 63×/1.4 oil-immersion lens with 2× digital zoom at a step size of ∼0.1 μm, and the total thickness was ≤100 μm. The 126× images were taken with 2048 × 2048 pixels. Step sizes were determined according to the optimized setting for each objective lens.

### Image analysis and quantification

ImageJ/FIJI (version 1.53t; National Institutes of Health) software was used for histologic analysis. The TCA signal intensities were used to distinguish barrels and septa areas in *post hoc* analysis with confocal images (taken by a 10× lens with 2× zoom). TCA cluster boundaries were defined as barrel edges. *In vivo*-imaged L4 neurons in barrels were identified in confocal images according to their relative positions with each other and their dendritic patterns. The distance of neurons to barrel edges was defined as the distance from the center of the cell body to the nearest barrel edge, and within 20 μm were classified as edge-located neurons. Neurons located barrel inside and distances to the barrel edge >20 μm were classified as barrel-center neurons and not included in the analysis of the current study.

Acquired 3D neuron images from two-photon microscopy were analyzed using the IMARIS Filament Tracer (version 9.5.1; Bitplane) software to reconstruct dendritic patterns and quantify basal dendritic morphologies. To keep the accuracy and efficiency of data analysis, we selected *in vivo*-imaged neurons for analysis with the following three criteria: (1) basal dendritic morphologies were clearly visible both in *in vivo* imaging (all nine imaging sessions) and *post hoc* confocal imaging; (2) neurons were located on the edges of main barrels (rows A–E, arcs 1–5); and (3) neurons were surrounded by two (or more) main barrels. In total, 19 neurons from three pups satisfied the above criteria. The same “dendritic trees” and “dendritic segments” were identified from different imaging sessions. A dendritic tree is an extension originating from the cell body. A dendritic segment was defined as a segment between two branching points or between a branching point and the origin of the dendrite (or the distal end). A primitive dendritic tree with no branches is also a dendritic segment. Newly formed and eliminated dendrite segments were detected by comparing the dendrite morphologies of the same neuron between two consecutive sessions. A 180° barrel boundary was used to separate barrel inner and outer sides as in our previous study (Nakazawa et al., 2018). When more than half the length of a dendritic segment of a basal dendrite was located in the barrel inner side, it was defined as an “inner segment.” The orientation bias index (OBI) is the total inner segment length/total basal dendritic length. The average OBI of nine imaging sessions from the same neuron was used as the OBI of the neuron.

For the quantification of basal dendritic tree and tip dynamics, the number of emerged, eliminated, and transient basal dendritic trees/tips from each neuron in 8 h of imaging was calculated. These dynamic events of dendritic tips were normalized with the total basal dendritic length or the total inner or outer segment length (no./millimeter) of their corresponding neuron. We classified length changes (in micrometers) of “tip segments” into the following three subgroups: R (length change, <−3 μm), E (length change, >3 μm), and stable (length change within 3 μm), as previously reported (Mizuno et al., 2014). A tip segment is defined as a dendritic segment whose one end is a dendritic tip. A primitive dendritic tree with no branches is also a tip segment. For the analysis of dynamics in 2 consecutive hours, tip segments that were continuously existing (no formation or elimination) were selected.

To analyze the correlation of dendrite tip morphologies with its dynamics, we first investigated all basal dendritic tips of 19 neurons (h0 to h7 of imaging session) and identified 64 tips that were apparently thick. Most of these thick parts of the dendritic tips were between 5 and 10 μm in length. Therefore, we used the most distal 5 μm part of the dendritic tip as the T part, skipped the adjacent 5 μm, and then used the subsequent 5 μm as the shaft (S) part. By excluding 5 μm of the intermediate area from the analyses, we avoided overlaps between the T and S. Regions of interest (ROIs) of T and S were then determined based on the mRFP signals at the resolution of the single-pixel level. Initially, we picked out 126 tips that showed tip morphologies distinguishable from their surrounding environment with a segment length >15 μm and had no overlapping with other dendrites or imaging noise. Seventy-nine of 126 tips were continuously presented, without branching or branch elimination, in two consecutive sessions [between 1 h before (Time −1) and 1 h after (Time +1) of the tip thickness index (TTI)-checking session (Time 0)]. The mRFP signal intensity (gray value) of ROIs was analyzed with the ImageJ/FIJI software: TTI of the dendritic tip = (gray value of T)/(gray value of S). We compared TTI and the length change (in micrometers) between Time −1 and Time +1.

### Statistical analysis

The quantification of the basal dendritic dynamics of L4 neurons was done with Microsoft Excel (Microsoft 365). The statistical analyses of dynamics comparisons were performed with GraphPad Prism (version 9.3.1). The significance of the comparisons was assessed by the Mann–Whitney test, Wilcoxon matched-pairs signed-rank test, or paired *t* test. Values are given as the mean ± SE of the median. The asterisks in the figures indicate the following: *p < 0.05, **p < 0.01, ***p < 0.001, and ****p < 0.0001. When *p* > 0.05, it indicates ns.

### Reanalysis of the data of previous longitudinal *in vivo* imaging

We reanalyzed the P4 *in vivo* imaging data of OBIs from our previous study (Nakazawa et al., 2018). We compared the OBI difference of L4 neurons at P4_M_ (12 P.M.) and P4_L_ (8 P.M.) since that time window fits with the time at which we performed *in vivo* imaging in the current study (1 P.M. to 9 P.M. at P4). We also analyzed the OBI differences between SS and star pyramid (SP) neurons at P4 (the average OBI of P4_M_ and P4_L_; see Table 2).

## Results

### *In vivo* imaging of developing dendrites with improved spatiotemporal resolution

**Figure 1.**
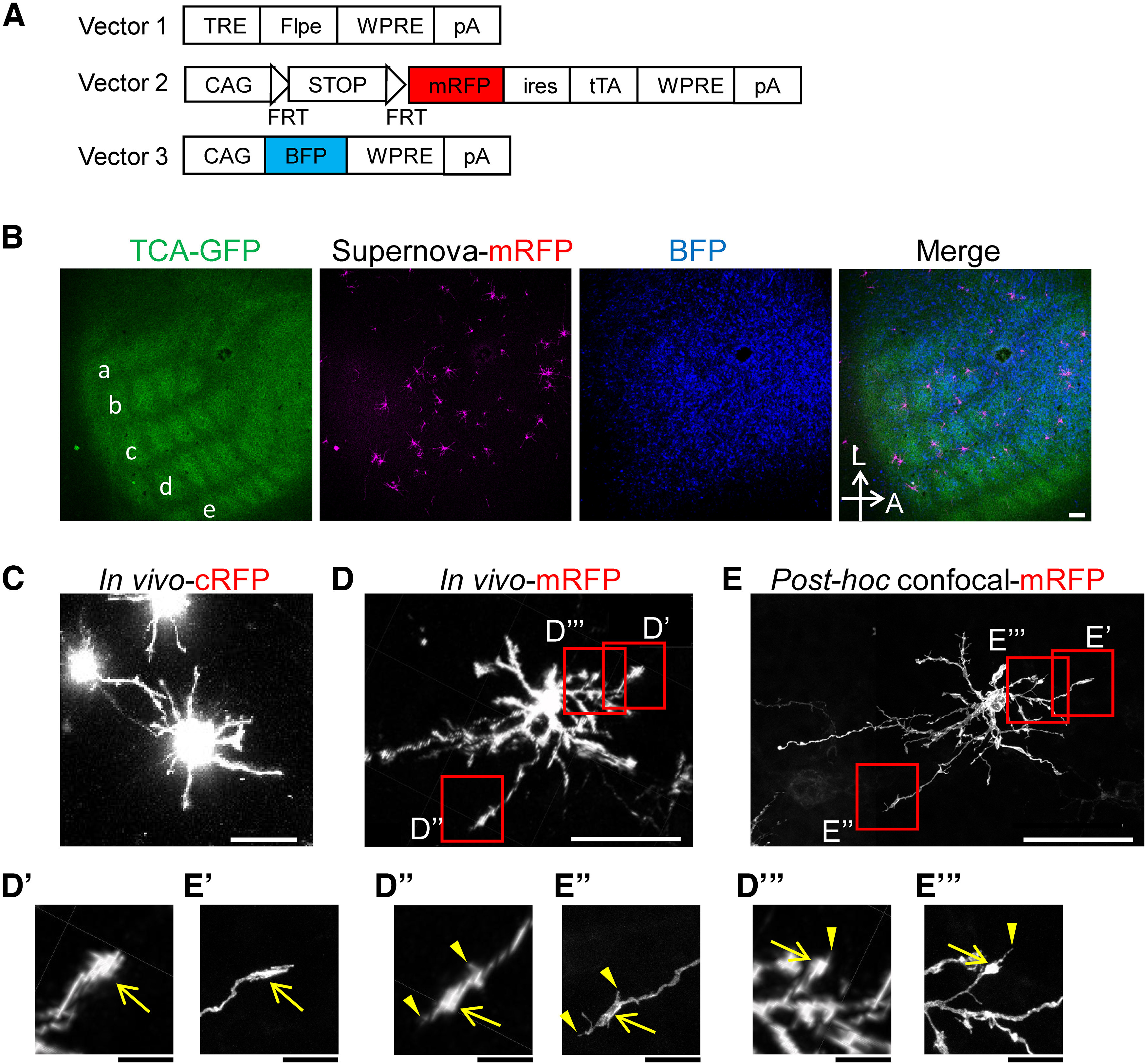
Improvement in the spatial resolution of cortical neuron *in vivo* imaging. ***A***, The Supernova membrane-bound RFP (mRFP) vector set (Vector 1 and Vector 2) used for IUE-based sparse labeling of barrel cortex L4 neurons. Vector 3 was used to identify mice in which neurons in the barrel field were appropriately labeled before the cranial window surgery because dense BFP, but not Supernova-mRFP, labeling by Vector 3 was detectable over the skull. ***B***, Tangential stacks of Supernova-mRFP-labeled L4 neurons in the TCA-GFP Tg mouse at P4. Left to right, the barrel map visualized with TCA-GFP Tg labeling, Supernova-mRFP-labeled L4 neurons, BFP-labeled L4 neurons, and the merged image. A, Anterior; L, lateral. Scale bar, 100 μm. ***C***, *In vivo* images of an L4 neuron labeled with Supernova-cRFP at P4 (*z*-stacked). Scale bar, 50 μm. ***D***, Tangential stacks of *in vivo* images of a Supernova-mRFP-labeled L4 neuron. Scale bar, 50 μm. ***D*′–*D*″′**, Higher-magnification images of the red box regions in ***D*** showed growth cone-like structures (arrows) and filopodium-like protrusions (arrowheads). Scale bar, 10 μm. ***E***, Tangential stacks of a high-magnification confocal image of the neuron shown in ***D***. Images were taken by a 63× lens with a 2× zoom. Scale bar, 50 μm. ***E*′–*E*′′′**, Higher-magnification images of corresponding areas shown in ***D*′–*D*″′**. The growth cone-like and filopodium-like morphologies were confirmed (arrows and arrowheads, respectively). Scale bar, 10 μm.

To visualize the dendritic morphology of neonatal cortical neurons *in vivo*, we used the IUE-based Supernova system (Mizuno et al., 2014; Luo et al., 2016), which labels cortical neurons sparsely and brightly for improved signal to background (Fig. 1*A*,*B*). As a fluorescent label, in our previous studies we used regular RFP (cRFP; Mizuno et al., 2014; Nakazawa et al., 2018), but the much stronger fluorescence in the cell body than dendrites (Fig. 1*C*) necessitated multiple tracings of the same dendrite at different image contrast settings for complete and accurate reconstruction. To solve this problem and facilitate faster dendrite reconstruction, in the current study we used mRFP (Liu et al., 1994; Moriyoshi et al., 1996), which reduced somatic relative to dendritic emission and enabled clearer visualization of dendritic morphologies *in vivo* (Fig. 1*D*). This enhanced accuracy and efficiency even enabled detailed detection of dendritic tips, including growth cone-like and filopodium-like structures (Fig. 1*D*′–*D*″′), which were subsequently confirmed by *post hoc* high-magnification confocal imaging in brain slices (Fig. 1*E*′–*E*′″).

**Figure 2.**
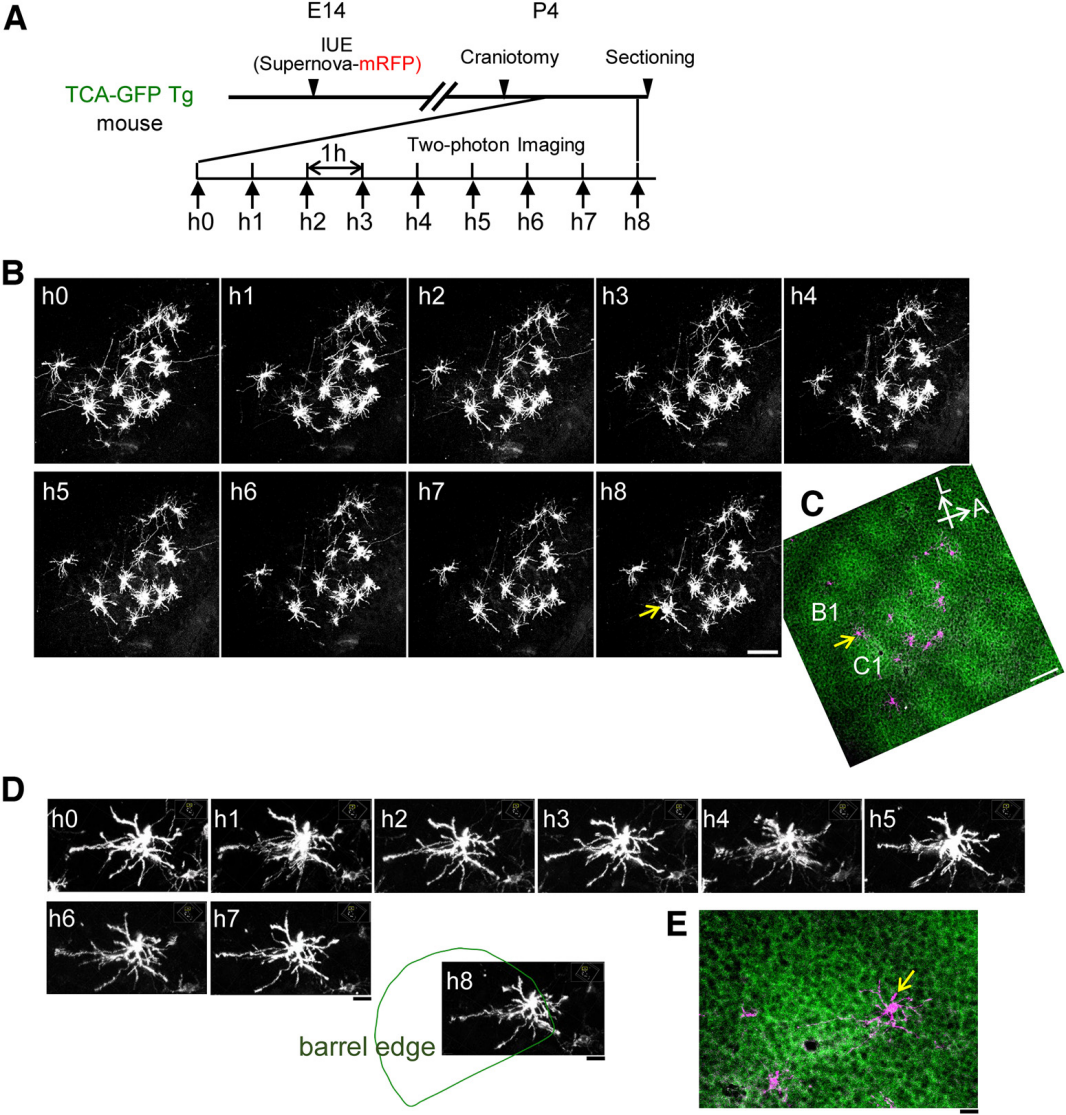
One-hour interval *in vivo* time-lapse imaging of L4 neurons in the neonatal barrel cortex. ***A***, Experimental design of time-lapse *in vivo* imaging. TCA-GFP Tg mice were used for visualizing the barrel map. L4 neurons in the TCA-GFP mouse barrel cortex were sparsely labeled with Supernova-mRFP via IUE at E14. The cranial window was made in the morning on P4, and the 1 h interval *in vivo* imaging started 1 h after the surgery. In every imaging session, the pup for *in vivo* imaging was head fixed to the imaging stage under a two-photon microscope with light anesthesia. The brain of the *in vivo* imaged mouse was collected after 9 (h0 to h8) imaging sessions, and then fixed. Tangential sections were obtained for *post hoc* confocal analyses. ***B***, An example of 1 h interval *in vivo* imaging of Supernova-mRFP-labeled L4 neurons at P4; *z*-stacked images from the top view. Scale bar, 100 μm. ***C***, The *post hoc* confocal image (*z* stacked) of the tangential section of the same area in ***B***. The neuron pointed with a yellow arrow in ***C*** is the same neuron pointed in the h8 session of ***B***. The same neurons were identified by their relative positions with each other and dendritic patterns. The *in vivo* imaged neurons were located on C1−C4 barrels in this example mouse. The sample orientations in ***B*** and ***C*** were similar. A, Anterior; L, lateral. Scale bar, 100 μm. ***D***, An example of time-lapse images of dendritic morphologies of an L4 neuron in 8 h of imaging sessions. Top views of an L4 neuron (*z*-stacked), which was located at the edge of the C1 barrel. Snapshots were taken from the 3D reconstruction software Imaris. The neuron was rotated to the same angle and at the same magnification from 9 imaging sessions. The green line in the h8 imaging session showed the C1 barrel edge. Scale bar, 20 μm. ***E***, The corresponding confocal image (*z*-stacked) of the neuron (yellow arrow) shown in ***D*** at a similar angle and magnification. An example neuron is located at the C1 barrel edge. Scale bar, 20 μm.

To visualize the barrel map, we used TCA-GFP Tg mice (Mizuno et al., 2014). We labeled L4 neurons in the barrel cortex of these mice with the IUE-based Supernova-mRFP. We then performed craniotomy at P4 (morning) and conducted hourly time-lapse imaging for a total of 8 h (or nine imaging sessions, h0 to h8; Fig. 2*A*,*B*). Brain samples were then collected for *post hoc* histologic analysis (Fig. 2*C*). For quantitative analysis of dendritic dynamics, we considered only L4 neurons located on barrel edges with clear basal dendritic patterns for all nine imaging sessions (see Materials and Methods). Tangential views of an example neuron from h0 to h8 sessions and *post hoc* histologic imaging are shown in Figure 2, *D* and *E*, respectively. In total, we obtained dendritic images from 19 neurons (Table 1) in three mice satisfying these criteria.

**Table 1:** Summary of 19 neurons that were used for statistical analyses

Neuron ID	Barrel	Distance	OBI	Type	AD	AD length (μm)
#1*	C1	12.8	0.689	High	−	29.7
#2	B2	10.6	0.415	Low	+	157.6
#3	C3	2.7	0.464	Low	+	190.1
#4	C2	9.9	0.475	Low	+	178.0
#5	C2	0	0.386	Low	+	486.8
#6	C4	5.8	0.747	High	+	234.6
#7	C3	2.4	0.714	High	−	0
#8	B1	11.8	0.666	High	+	187.4
#9	B2	11.7	0.616	High	+	189.6
#10	B4	3.3	0.545	Medium	+	267.1
#11	C5	4.2	0.562	Medium	+	124.7
#12	B3	9.9	0.465	Low	+	435.8
#13	B3	19.7	0.757	High	−	0
#14	B3	2.7	0.454	Low	+	679.8
#15	B2	16.1	0.693	High	−	0
#16	C2	1.7	0.644	High	+	225.7
#17	B2	7.4	0.381	Low	+	229.8
#18	C2	19.3	0.681	High	+	447.5
#19	C2	14.0	0.367	Low	+	207.8

Barrel, Barrel column that the neuron belongs to; Distance, the distance (in μm) from the center of cell body to the barrel edge; OBI, Mean OBI (see Materials and Methods) of 9 imaging sessions of the neuron; High, high-OBI neuron, whose OBI was >0.6; Low, low-OBI neuron, whose OBI was <0.5; Medium, neurons whose OBI values were >0.5 and <0.6 were not included in either high-OBI or low-OBI groups; AD, apical dendrite; +, −, mean presence and absence of the apical dendrite, respectively.

*Note that Neuron #1 has only an extremely short (∼30 μm) apical dendrite.

**Figure 3.**
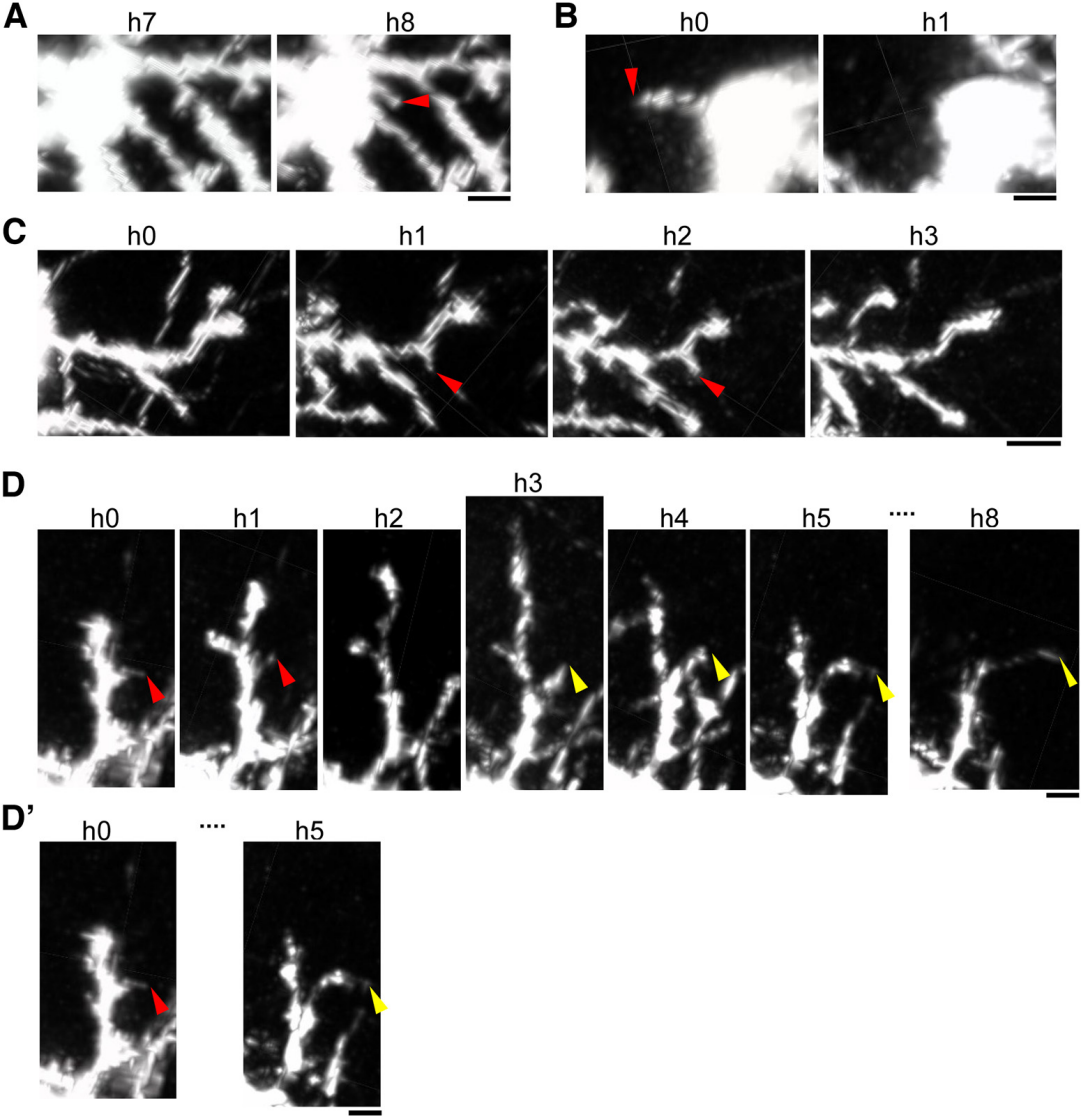
Detection of rapid dendritic dynamics with improved spatiotemporal resolution *in vivo* imaging. ***A***, A basal dendritic tree (arrowhead) that newly emerged between h7 (left) and h8 (right) sessions. Snapshot images taken in the Imaris software. Scale bar, 5 μm. ***B***, A basal dendritic tree that disappeared between h0 (left) and h1 (right) sessions. Snapshot images taken in the Imaris software. Scale bar, 5 μm. ***C***, A transient basal dendritic branch (arrowheads) emerged between h0 and h1 sessions and disappeared between h2 and h3 sessions. Scale bar, 10 μm. ***D***, ***D*′**. After a branch (red arrowheads) was eliminated between h1 and h2 sessions, another branch (yellow arrowheads) emerged at a similar position between h2 and h3 sessions (***D***); it is highly likely that these two branches (red and yellow arrowheads) are recognized as an identical dendrite if only h0 and h5 images are available (***D*′**). Snapshot images taken in the Imaris software. Scale bar, 5 μm. Examples in ***A–C***, ***D***, and ***D*′** are from Neuron 1 and Neuron 3 (Table 1), respectively.

This improved spatiotemporal resolution revealed rapid changes in dendrite morphology, including the emergence (Fig. 3*A*,*C*,*D*) and elimination (Fig. 3*B–D*) of dendritic trees and branches between two consecutive imaging sessions. These rapid dendrite dynamics detected with hourly imaging were difficult to detect using our previous 8 h interval imaging strategy. For example, in the case of Figure 3*C*, a dendritic branch that emerged between the first and second sessions (h0 and h1) disappeared between the h2 and h3 sessions. This kind of transient branches were likely missed by our previous 8 h interval imaging. In the case of Figure 3*D*, after a branch was eliminated between the h1 and h2 sessions, another branch emerged at a similar position between h2 and h3 sessions. It is highly likely that these two branches at similar locations were recognized as the same branch by lower time-resolution imaging (Fig. 3*D*′). Thus, the high spatiotemporal resolution achieved using mRFP and shorter acquisition interval in the current study allowed us to conduct more comprehensive mechanistic analyses.

### Three-dimensional reconstruction of L4 neuron dendritic patterns

**Figure 4.**
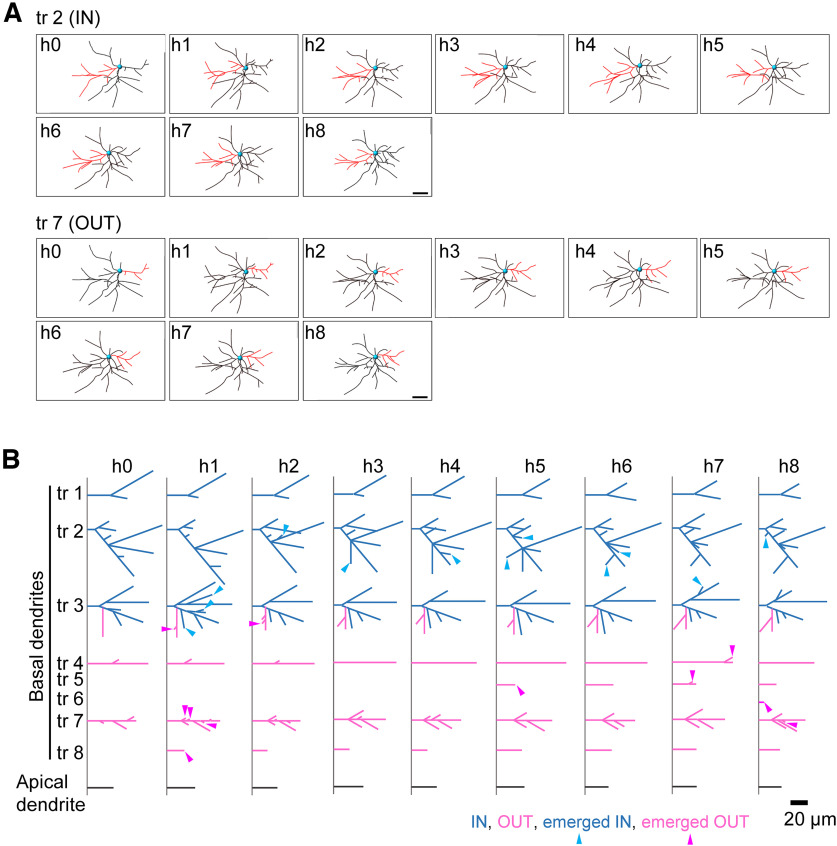
Reconstruction of dendritic patterns imaged *in vivo*. ***A***, A two-dimensional view of 3D reconstructed dendritic morphology from the neuron shown in Figure 2*D*. Representative basal dendritic trees (tr 2 and tr 7) that originated from the barrel-side half (IN) of the cell body and the other half (OUT), respectively, are labeled in red. Scale bars, 20 μm. ***B***, A schematic that enables simple tracking of dendritic pattern changes during imaging sessions. The representative schematic for the example neuron in ***A*** is shown. Imaging session numbers (h0 to h8) are shown on the top. The same basal dendritic trees (tr 1−8) were arranged in the same rows in schematics. The same dendritic segments from different imaging sessions were placed at the same angles. Individual dendritic segments were classified into barrel-inner (IN; cyan) and barrel-outer (OUT; magenta) according to their location. The newly emerged inner and outer branches (and trees) were marked with cyan and magenta arrowheads, respectively. The lengths of individual dendritic trees and segments are approximately proportional. Gray lines, Apical dendrite. Scale bar, 20 μm.

To reveal the detailed dendritic dynamics of barrel cortex L4 neurons at P4, we reconstructed the 3D morphology of all dendrites (basal and apical dendrites) from 19 neurons (the example neuron in Fig. 4 is the same neuron as shown in Fig. 2*D*). After tracing and reconstructing dendritic patterns of the same neuron from the nine imaging sessions, we simplified the temporal patterns by drawing schematics for more efficient evaluation of morphologic changes (Fig. 4).

We identified the same dendritic trees, which are extensions originating from the cell body, across imaging sessions by comparing morphologies in the 3D view (Fig. 4*A*). Then, tree IDs (e.g., tr X) were assigned to the individual basal dendritic trees of each neuron (Fig. 4*B*). The same trees from each imaging session were arranged in the same rows in the schematics (left to right, h0 to h8 imaging sessions). The same dendritic segments (see Materials and Methods) were also identified from different imaging sessions and placed at the same angles. We then classified individual dendritic segments into barrel-inner and barrel-outer according to location as previously described (Nakazawa et al., 2018). Inner and outer dendritic segments accounted for all or the majority of segments belonging to the barrel-inner and barrel-outer halves, respectively. If a dendritic tree extends along the barrel border, it is possible that it includes both inner and outer dendritic segments (Fig. 4*B*, tr 3).

By comparing the same dendritic trees and dendritic segments in schematics, we were able to easily identify newly emerged (arrowheads in schematics) and eliminated dendritic trees and branches. These schematics were useful for characterizing detailed changes in dendritic patterns during the 8 h of *in vivo* imaging.

### Basal dendritic dynamics of L4 neurons in neonatal barrel cortex

**Figure 5.**
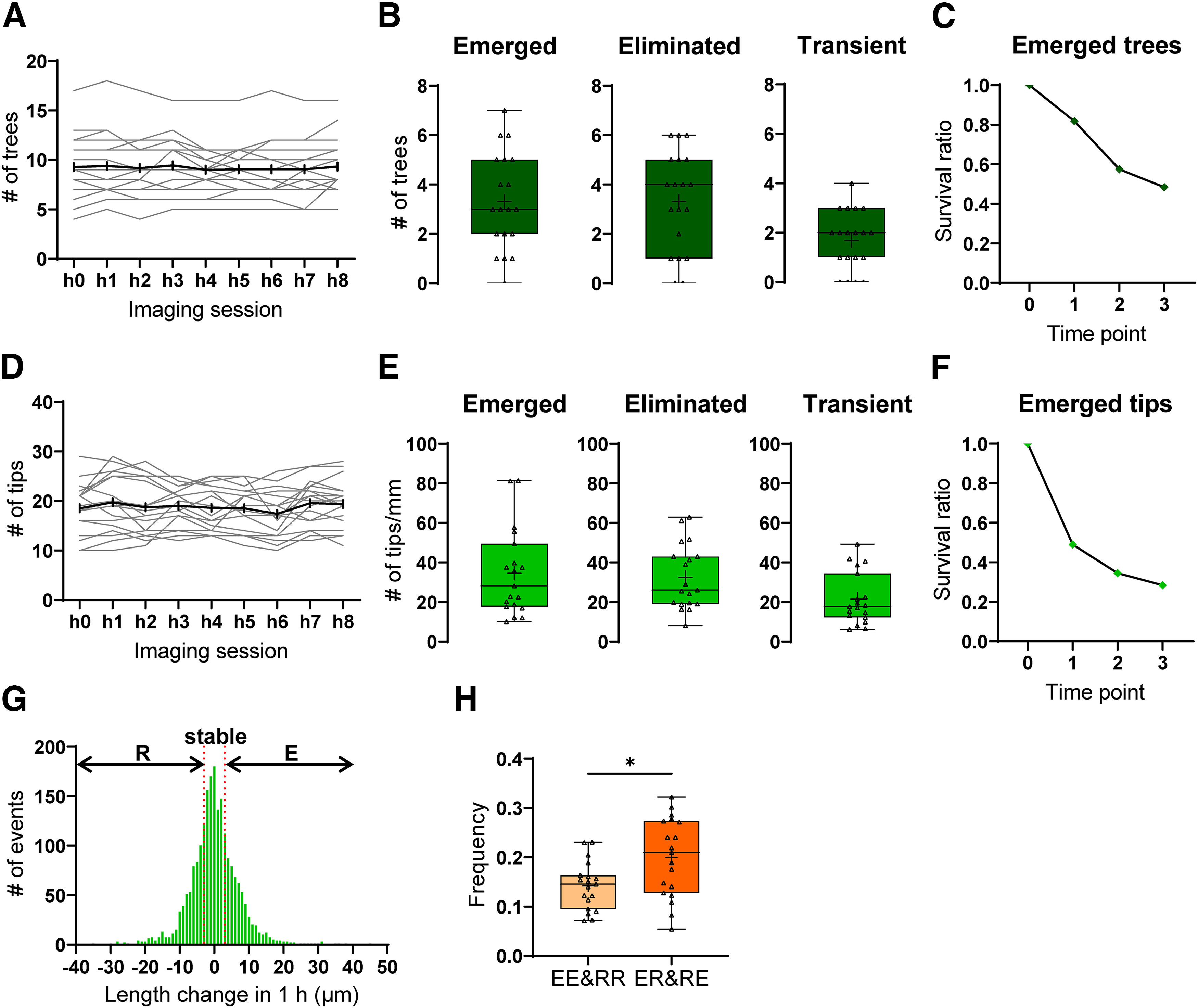
General properties of the dendritic dynamics of L4 neurons at P4. ***A***, Changes in basal dendritic tree numbers from each neuron during 8 h of imaging. The gray and black lines represent data of individual neurons and the average, respectively. *n* = 19 neurons, 3 mice. ***B***, Numbers of emerged, eliminated, and transient trees from each neuron in 8 h of imaging. +, The mean value; horizontal line in the boxplot, the median. Each dot represents one neuron. *n* = 19 neurons, 3 mice. ***C***, Survival ratio of newly emerged trees. In this analysis, only dendritic trees that were first detected at h1, h2, h3, h4, or h5 imaging sessions were used. The imaging session at which the tree was first detected was defined as the time point 0 (*n* = 33 trees); 27 (81.8%), 19 (57.6%), and 16 (48.5%) of the 33 trees were still present at time points 1, 2, and 3, respectively. *n* = 16 neurons, 2 mice. Three of 19 *in vivo* imaged neurons have no trees that emerged between h1 and h5 sessions. ***D***, Changes in dendritic tip numbers from each neuron during 8 h of imaging. The gray lines and black lines represent data of individual neurons and the average, respectively. *n* = 19 neurons, 3 mice. ***E***, Numbers of emerged, eliminated, and transient tips (per mm) from each neuron in 8 h of imaging. +, The mean value, and the line in the boxplot represents the median value. Each dot represents one neuron. *n* = 19 neurons, 3 mice. ***F***, Survival ratio of the newly emerged dendritic tips. Only dendritic tips that emerged between h1 to h5 of the *in vivo* imaging sessions were used. At the time point 0, *n* = 214 tips; 105 (49.1%), 74 (34.6%), and 61 (28.5%) of 214 dendritic tips were still present at time points 1, 2, and 3, respectively. *n* = 19 neurons, 3 mice. ***G***, Histogram of dendritic tip length changes in 1 h. The *x*-axis shows the length change (μm), and the *y*-axis shows the event number of corresponding length changes. Length changes >3 μm, smaller than −3 μm, and between −3 and +3 μm were classified as E (28.1%), R (28.5%), and stable (S; 43.4%), respectively. *n* = 2089 dendritic tips (from 19 neurons, 3 mice). ***H***, Frequencies of tip behavior in which individual tips continued to elongate or retract in 2 consecutive imaging sessions (EE and RR) and those of tip behavior in which dendritic tips changed the motility direction from E to R or R to E (ER and RE) were compared. Other behaviors (ES, RS, SE, SR, and SS) were excluded from comparison. +, The mean value and the horizontal line in the boxplot represents the median. Each dot represents one neuron. *p* = 0.022, Mann–Whitney test. *n* = 19 neurons, 3 mice.

We first analyzed the general properties of dendritic tree dynamics *in vivo*. The total number of basal dendritic trees per neuron was rather stable during the 8 h of imaging (Fig. 5*A*). To investigate the dynamics of L4 neuron dendritic trees systematically, we quantified emerged, eliminated, and transiently appearing (emerged and then eliminated within 8 h) trees across the nine imaging sessions. In the temporal window, 3.32 ± 0.45 trees emerged and 3.32 ± 0.46 were eliminated. Notably, a substantial number (1.68 ± 0.28) were transient (Fig. 5*B*). Further, newly emerged trees were often short lived, and ∼20% (6 of 33) disappeared by the next imaging session (Fig. 5*C*), underscoring the importance of the improved temporal resolution.

Next, we analyzed dendritic tip dynamics of L4 neurons. The total number of basal dendritic tips from each neuron did not change substantially over 8 h (Fig. 5*D*). We then normalized the numbers of basal dendritic tips that emerged, were eliminated, or appeared transiently in 8 h by the total basal dendritic length (see Materials and Methods) and again found similar densities of emergence and elimination (34.62 ± 5.01 vs 32.44 ± 3.73/mm) over 8 h (Fig. 5*E*). There were 21.50 ± 3.00/mm transient tips, and about half (109 of 214) of the newly emerged tips disappeared by the next imaging session (Fig. 5*F*).

We then grouped basal dendritic tips into three categories, R (shortened >3 μm), E (extended >3 μm), and S (length changes <3 μm; see Materials and Methods). Approximately 28% of tips retracted during the time window, 28% elongated, and 43% were stable (Fig. 5*G*). An important parameter of dendritic dynamics is how often dendritic tips switch between E and R behavior. We quantified these events for two consecutive imaging sessions as follows. When a tip continued to elongate across two consecutive sessions, it was categorized as Elongation-Elongation (EE), and when a tip became shorter across 2 consecutive hours, it was categorized as Retraction-Retraction (RR). Further, when a tip was elongated at one measurement time and was shorter at the next, it was categorized as Elongation-Retraction (ER) and vice versa. The overall frequency of switching events (ER and RE) was higher than the frequency of stability (EE and RR) over two consecutive sessions (Fig. 5*H*). This result further highlights the highly dynamic nature of L4 neuron basal dendrites at P4.

### Comparison of short-term dynamics between the inner and outer basal dendrites

**Figure 6.**
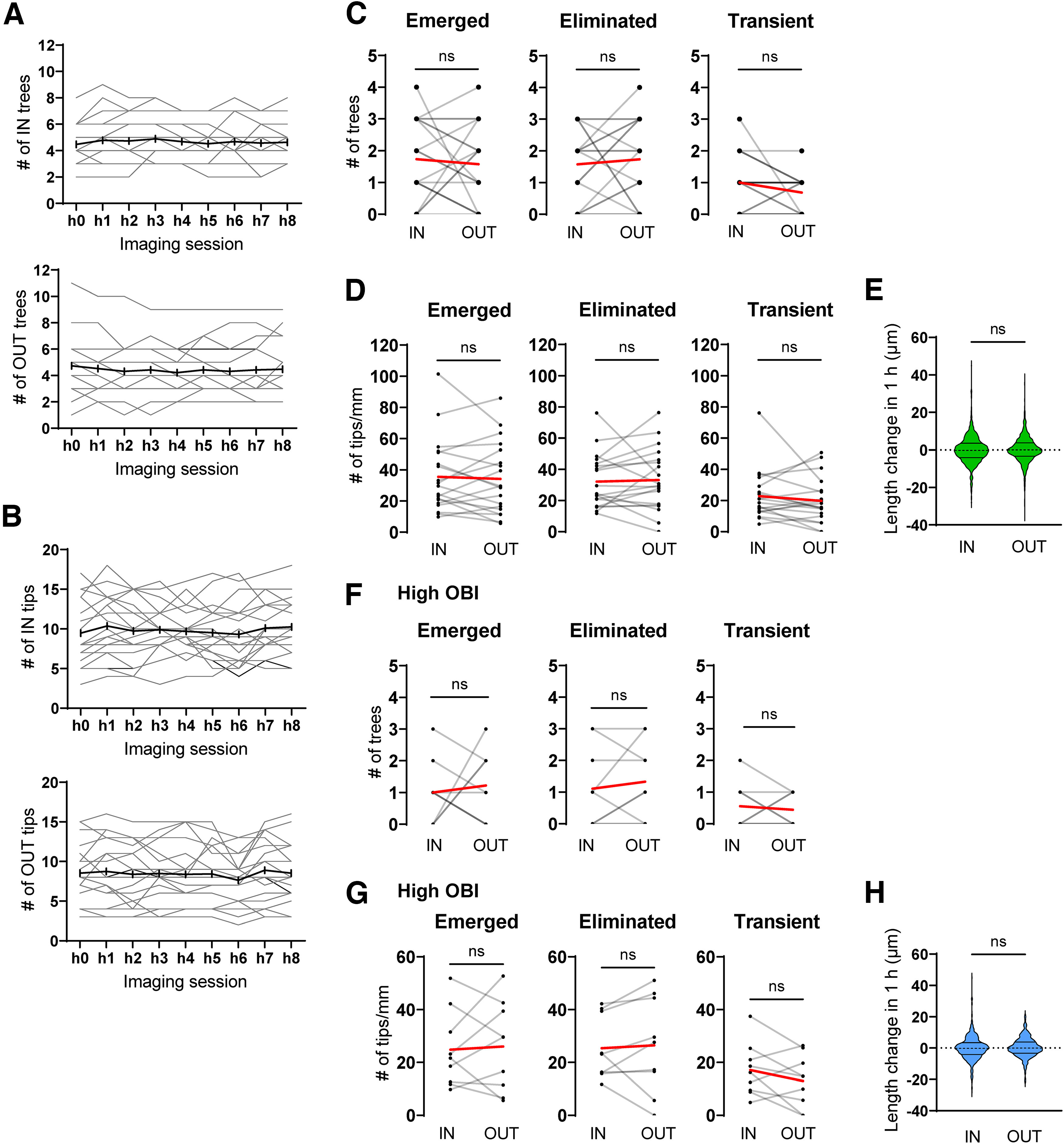
Comparison of dynamic properties between the inner and outer dendrites. ***A***, ***B***, Changes in the numbers of inner (IN) and outer (OUT) trees (***A***) and tips (***B***) from each neuron across 9 imaging sessions. Gray lines, Data for individual neurons; black lines, Average of all neurons. *n* = 19 neurons, 3 mice. ***C***, There were no significant differences between IN and OUT trees in the numbers of emerged, eliminated, and transient trees. *p* = 0.796, 0.556, and 0.234, respectively; Wilcoxon matched-pairs signed-rank tests; *n* = 19 neurons, 3 mice. Red lines, Average. ***D***, There were no significant differences between IN and OUT tips in the numbers of emerged, eliminated, and transient tips (per mm basal dendritic length; *p* = 0.738, 0.568, and 0.441, respectively; Wilcoxon matched-pairs signed-rank tests; *n* = 19 neurons, 3 mice). The dendritic tip numbers were normalized with the IN or OUT basal dendritic length of the neuron (average length from 9 imaging sessions). Red lines, Average. ***E***, There were no significant differences between IN and OUT dendritic tips in 1 h length changes (μm). *p* = 0.304; Mann–Whitney test; IN tips, *n* = 945; OUT tips, *n* = 852 (from 19 neurons, 3 mice). ***F***, There were no significant differences between IN and OUT basal dendritic trees of high-OBI neurons in the numbers of emerged, eliminated, and transient trees. *p* = 0.648, 0.750, and >0.999, respectively; Wilcoxon matched-pairs signed-rank tests. *n* = 9 high-OBI neurons, 3 mice. Red lines, Average. ***G***, There were no significant differences between IN and OUT dendritic tips of high-OBI neurons in the numbers of emerged, eliminated, and transient tips (per mm IN or OUT basal dendritic length; *p* = 0.820, 0.570, and 0.250, respectively; Wilcoxon matched-pairs signed-rank tests). *n* = 9 high-OBI neurons, 3 mice. Red lines, Average. ***H***, There were no significant differences between IN and OUT tips of high-OBI neurons in 1 h length changes (μm). *p* = 0.260; Mann–Whitney test; high-IN tips, *n* = 568; high-OUT tips, *n* = 340 (from 9 high-OBI neurons, 3 mice).

Although L4 neurons in barrel cortex have both inner and outer basal dendrites, these neurons receive inputs primarily onto inner basal dendrites because TCAs densely innervate the barrel center but not the septa (Fig. 2*C*). Dendritic refinement largely relies on TCA inputs (Harris and Woolsey, 1981; Narboux-Nême et al., 2012; Li et al., 2013; Mizuno et al., 2018; Nakazawa et al., 2018, 2020), so the spatial bias of TCA inputs may drive dendritic dynamics. The numbers of inner dendritic trees and tips did not change markedly during 8 h of imaging (Fig. 6*A*,*B*). Similarly, the number of outer dendritic trees and tips did not change substantially (Fig. 6*A*,*B*). Moreover, the numbers of newly emerged, eliminated, and transient trees were similar between the inner and outer portions of barrels over 8 h (Fig. 6*C*). The numbers of dendritic tips (per total inner or outer dendritic length) that emerged, were eliminated, and transiently appeared within 8 h did not differ significantly between inner and outer dendritic fields (Fig. 6*D*). Furthermore, the change in tip length across two consecutive imaging sessions was also similar between inner and outer fields (Fig. 6*E*). Thus, basal dendritic trees and tips demonstrated comparable short-term dynamics between inner and outer dendrites of barrel cortex L4 neurons at P4.

In the mouse barrel cortex L4, excitatory neurons are classified as SS or SP neurons. Our IUE-based Supernova labels both types of neurons, although the majority of labeled neurons are of the SS type (Nakazawa et al., 2018). The barrel center-oriented asymmetric basal dendritic pattern is a characteristic of SS neurons but not of SP neurons, suggesting potential differences in short-term dynamics between inner and outer basal dendrites, especially those of SS neurons. In the adult brain, SP neurons are distinguished by the presence of apical dendrites, which are lacking in SS neurons (Staiger et al., 2004; Scala et al., 2019). On the other hand, the majority of SS neurons at P4 still have the apical dendrite (Callaway and Borrell, 2011; Nakazawa et al., 2018) and thus are difficult to distinguish from SP neurons. However, our previous longitudinal *in vivo* imaging study conducted between P3 and P6 revealed that SS neurons exhibit a higher OBI (see Materials and Methods) than SP neurons even before the initiation of apical dendrite retraction (Nakazawa et al., 2018). Therefore, we here reevaluated all imaged neurons (14 SS neurons and 7 SP neurons) in our previous longitudinal imaging study and found that OBIs were significantly higher in SS than in SP neurons at P4 [0.417−0.858 (median = 0.730) vs 0.416−0.580 (median = 0.473); *p* = 0.006, Mann–Whitney test; Table 2). Based on this finding, we selected only neurons with high OBI (>0.600, 9 neurons) as prospective SS neurons from 19 neurons that were imaged in the current study (Table 1). Notably, four of nine high-OBI neurons had no apical dendrite or an extremely short (∼30 μm) apical dendrite, which is a characteristic of mature SS neurons. We then compared the dynamics between inner and outer basal dendrites of these high-OBI neurons and found that the numbers of trees and tips of basal dendrites that emerged, disappeared, or were transient were still similar between inner and outer dendrites. Thus, even within the high-OBI neuron group (i.e., prospective SS neurons), there were no differences in the dynamics between inner and outer dendrites (Fig. 6*F*,*G*). Furthermore, the length change in 1 h was also similar between inner and outer tips of high-OBI neurons (Fig. 6*H*).

**Table 2:** Reanalysis of neurons that were analyzed in previous longitudinal *in vivo* imaging

Neuron ID	OBI	Neuron type
#a	0.712	SS
#b	0.869	SS
#c	0.857	SS
#d	0.808	SS
#e	0.795	SS
#f	0.752	SS
#g	0.747	SS
#h	0.753	SS
#i	0.629	SS
#j	0.575	SS
#k	0.560	SS
#l	0.516	SS
#m	0.503	SS
#n	0.417	SS
#o	0.580	SP
#p	0.563	SP
#q	0.497	SP
#r	0.473	SP
#s	0.439	SP
#t	0.430	SP
#u	0.416	SP

**Figure 7.**
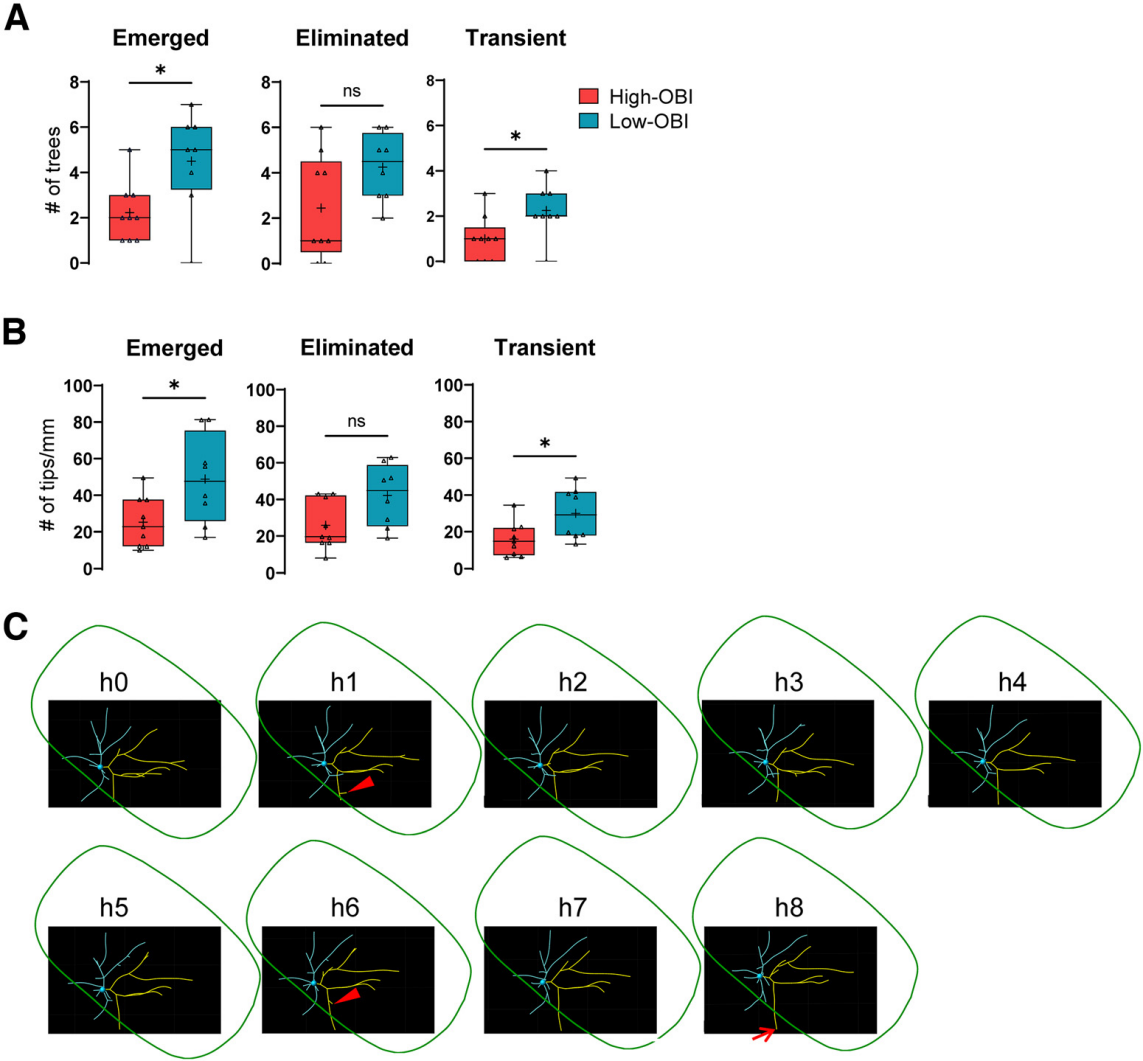
Comparison of dynamic properties of dendrites between high-OBI and low-OBI neurons. ***A***, Numbers of basal dendritic trees that emerged (*p* = 0.024), eliminated (*p* = 0.106), and were transient (*p* = 0.030) were compared between high-OBI and low-OBI neurons. +, The mean value; horizontal line in the boxplot, the median value. Each dot represents one neuron. Mann–Whitney tests; high-OBI neurons, *n* = 9; low-OBI neurons, *n* = 8. ***B***, Numbers of basal dendritic tips that emerged (*p* = 0.047), eliminated (*p* = 0.059), and were transient (*p* = 0.036) were compared between high-OBI and low-OBI neurons. The dendritic tip number was normalized with the total basal dendritic length of the neuron (average length from 9 imaging sessions). +, The mean value; horizontal line in the boxplot, the median value. Each dot represents one neuron. Mann–Whitney tests. high-OBI neurons, *n* = 9; low-OBI neurons, *n* = 8. ***C***, Reconstructed dendritic pattern for an example dendritic tree (yellow) from a high-OBI neuron, which is located near the barrel edge (green). An outward basal dendritic tip did not change its direction or start to retract at the barrel edge and elongated outward further (arrow at h8). Two inward tips were generated near the barrel-edge, but both were eliminated immediately (arrowheads at h1 and h6 sessions). Scale bar, 20 μm.

We also compared basal dendritic dynamics between high-OBI and low-OBI neurons (defined as <0.500, 8 of 19 neurons; Table 1), while neurons with intermediate OBIs (0.500−0.600, 2 neurons) were excluded. All low-OBI neurons had substantially longer apical dendrites, suggesting that these were SP neurons or less mature SS neurons. High-OBI neurons showed fewer newly emerged and transient basal dendritic trees, compared with low-OBI neurons (Fig. 7*A*). A similar tendency was observed in the number of eliminated dendritic trees, although the difference was not significant (*p* = 0.106). These results indicate that high-OBI neurons produce fewer trees than low-OBI neurons in a short time window. In other words, high-OBI neurons were more stable than low-OBI neurons. We next compared the dynamics of basal dendritic tips between high-OBI and low-OBI neurons. There were fewer emergent and transient dendritic tips (per millimeter) among high-OBI neurons compared with low-OBI neurons (Fig. 7*B*). The number of eliminated dendritic tips also showed a similar tendency for lower dynamics, although the difference was not significant (*p* = 0.059). It is likely that both of prospective SP and less mature SS neurons are categorized as low-OBI neurons at P4. In fact, in the reevaluation of our previous longitudinal imaging data, 1 of 14 SS neurons and 5 of 7 SP neurons showed low OBI (<0.500) at P4. Thus, these results from the current imaging may suggest that mature SS neurons have less dynamic basal dendrites than SP neurons and less mature SS neurons.

In addition to these quantitative analyses, we also carefully examined the behavior of individual basal dendritic trees located around barrel edges in high-OBI neurons, including whether the growth direction of dendritic tips is reoriented from outward to inward at the barrel edge and if basal dendrites form more branches inward than outward. Examination of all basal dendritic trees (43 trees from nine high-OBI neurons) expanded around the barrel edges revealed neither reorientation at the barrel edge nor more frequent inward orientation (Fig. 7*C*, example). There were no detectable differences in dynamics or turning direction between inner and outer tips. Both inner and outer tips demonstrated similar behaviors such as retraction, elongation, elimination, and emergence. These results suggest that basal dendritic asymmetry is generated not through directed outgrowth or reorientation of dendrites but through trial-and-error emergence, elongation, elimination, and retraction of dendritic branches and trees.

### The tip features of basal dendrites are correlated with behavior

**Figure 8.**
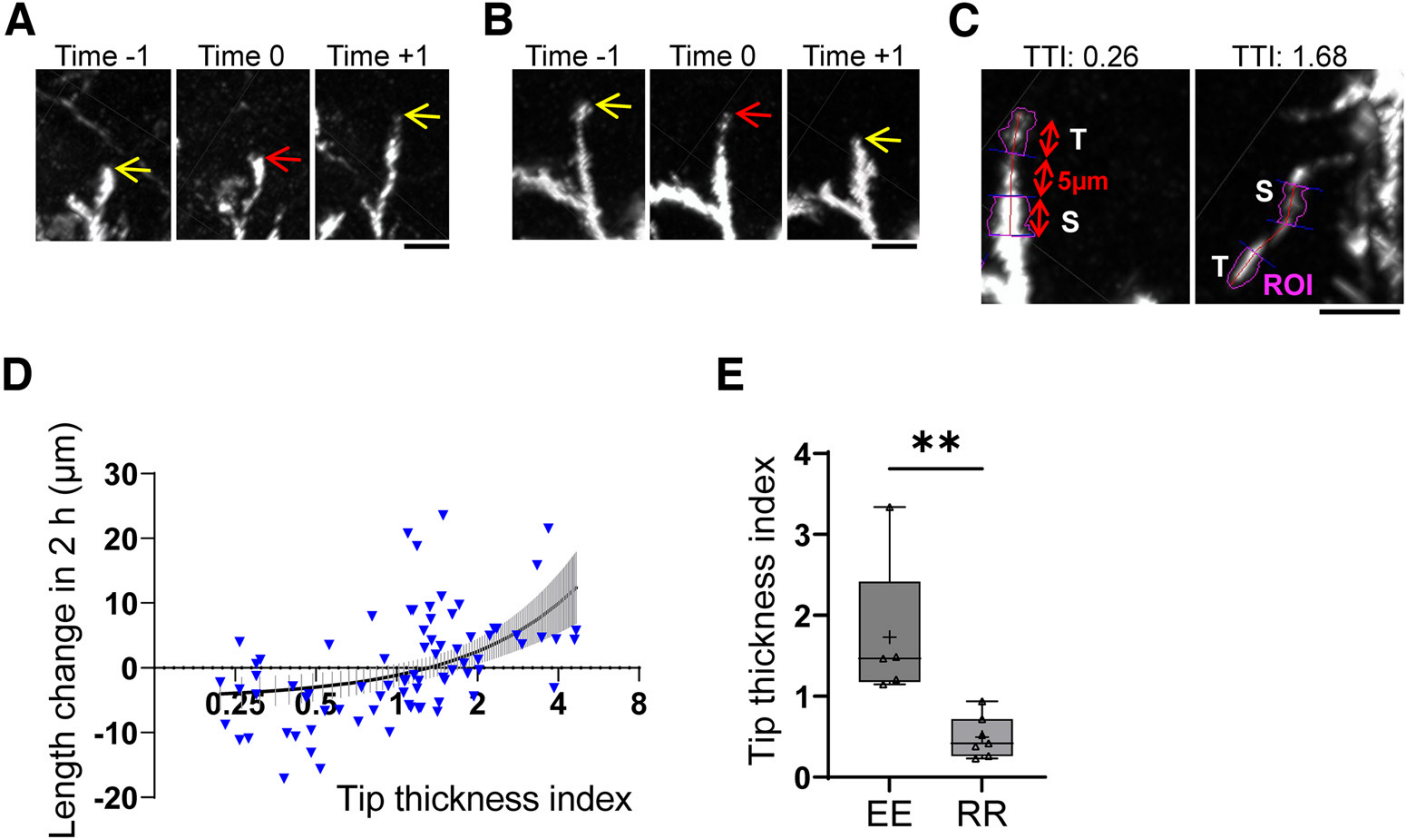
Dendrite tip features represent their behavior. ***A***, An example image of a dendritic branch (tip-segment) whose tip appeared thick at Time 0 (red arrow) was becoming longer and longer between the Time −1 and Time +1 sessions. Red and yellow arrows indicate the distal end of the branch. Snapshot images taken in the Imaris software. Scale bar, 10 μm. ***B***, An example image of a dendritic branch whose tip appeared thin at Time 0 (red arrow) was becoming shorter and shorter between the Time −1 and Time +1 sessions. Red and yellow arrows indicate the distal end of the branch. Snapshot images taken in the Imaris software. Scale bar, 10 μm. ***C***, Example diagrams of quantitative analysis for TTI. Left, An example for a thin tip, TTI = 0.26. Right, An example for a thick tip, TTI = 1.68. The distal part of the tip-segment was divided into 3 units: T, middle, and S. The length of each unit is 5 μm. ROIs (magenta areas) of T and S units were determined according to their morphologies. Snapshot images taken in the Imaris software. Scale bar, 10 μm. ***D***, Quantification of correlations of TTIs and length changes of tip-segments in 2 h (between Time −1 and Time +1). The *x*-axis is in log_2_ units. *y* = 3.689 * *x* – 4.826. The black curve represents the fitted curve, and the dashed line represents the 95% confidence interval. Each inverted triangle represents individual dendritic tips. *n* = 79 tips, from 14 neurons, 3 mice. ***E***, TTIs of tip-segments that continued to extend (EE) were compared with TTIs of tip-segments that continued to retract (RR) in 2 consecutive hours. In this analysis, TTIs were calculated from the middle time points of continuous elongation or retraction. +, The mean value; horizontal line in the boxplot, the median. Each dot represents one neuron. *p* = 0.003, Mann–Whitney test. *n* = 5 EE and 7 RR tip-segments, from 5 neurons, 2 mice.

Taking advantage of the high spatiotemporal resolution of our *in vivo* imaging system, we examined whether basal dendrite tip thickness represents its behavior. Indeed, elongating dendritic tips were often thicker (Fig. 8*A*, red arrow), while retracting tips were often thinner (Fig. 8*B*, red arrow). We analyzed these associations quantitatively by calculating correlations. For this analysis, we considered only dendritic tips with clear morphology and measured the mRFP signal intensity in the T and S parts to calculate a TTI by dividing T by S (Fig. 8*C*, examples; also see Materials and Methods). We found that TTI was positively correlated with tip segment length changes in 2 h (between 1 h before and 1 h after the tip thickness quantification; Fig. 8*D*). In other words, dendritic tips with smaller TTIs, which showed thinner tips in morphology, tended to be retracting while thicker tips with larger TTIs tended to be elongating. We also found that the continuously elongating (EE) tips in 2 consecutive hours had significantly larger TTIs than those of continuously retracting (RR) tips at the middle time point of extension or retraction (Fig. 8*E*).

**Figure 9.**
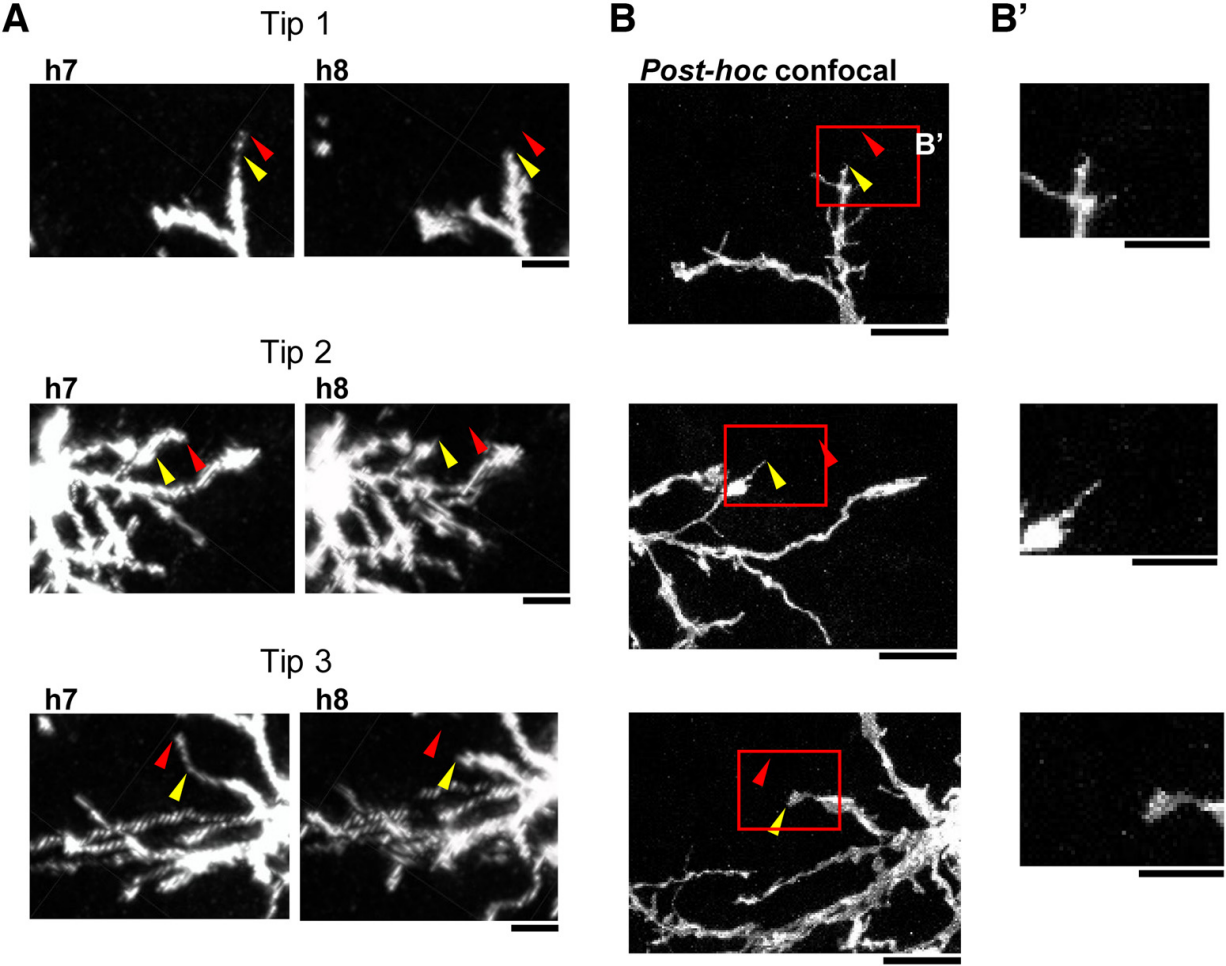
Observation of loci immediately after dendrite retraction. ***A***, Three representative basal dendritic tips retracted between h7 and h8 sessions. The red and yellow arrowheads show positions of basal dendritic tips in h7 and h8 sessions, respectively. Snapshot images taken in the Imaris software. Scale bars, 10 μm. ***B***, *Post hoc* (126×) confocal *z*-stack images corresponding to images in ***A***. The red and yellow arrowheads show positions of dendritic tips in h7 and h8 sessions, respectively. Brain slices, 100 μm thick. Scale bars, 10 μm. ***B*′**, The higher-magnification images of red box areas in ***B***. Scale bars, 5 μm.

In *Drosophila* larvae, dendritic pruning of class IV dendritic arborizing (C4da) sensory neurons is mediated by severing and degeneration, in which the proximal part of a dendrite is severed, and subsequently the parts of dendrites that are distal from the severed point are degenerated and disappeared (Kuo et al., 2005; Jan and Jan, 2010; Kanamori et al., 2013). To examine whether a similar severing/degeneration mechanism is involved in basal dendrite retraction of mouse neonatal cortical neurons, we focused on dendritic tips that were retracted between h7 and h8 sessions. We carefully observed both *in vivo* images of neurons at the h8 session and high-magnification confocal images of the same neurons in the *post hoc* fixed brain slices and examined whether there are debris-like structures at sites where the dendritic tip disappeared between the h7 and h8 sessions. No such debris-like structures were found in 41 instances of late tip disappearance (including 10 cases from the outer basal dendrites of high-OBI neurons; Fig. 9, examples). Thus, we found no evidence for the possibility that dendritic retraction in barrel cortex L4 neurons involves dendrite severing and degradation.

## Discussion

### Short-term dynamics of dendrites in the neocortex during developmental refinement

In the current study, we analyzed the short-term dendritic dynamics of L4 neurons in the mouse barrel cortex during neonatal development. *In vivo* imaging at 1 h intervals allowed us to precisely identify the same dendritic trees and branches across imaging sessions (Figs. 2-4), which were often difficult in previous 8 h interval imaging (Nakazawa et al., 2018). In addition to greater temporal resolution, this study used a membrane-bound RFP, which enabled the precise visualization of dendrite morphology *in vivo* without laborious contrast adjustments (Fig. 1). These improvements in spatiotemporal resolution allowed us to detect detailed morphologic changes at the level of “individual” dendritic trees and branches in the neonatal cortex (Figs. 3, 4). We found that many dendritic trees and branches (tip segments) emerged and were eliminated within 8 h (Fig. 5*A*–*F*). In addition, dendritic tips often changed direction from elongation to retraction or from retraction to elongation between two consecutive imaging sessions (Fig. 5*G*,*H*). Thus, basal dendrites of barrel cortex L4 neurons are highly dynamic during the refinement period in neonatal development.

Traditional studies of dendrite dynamics in the developing mammalian brain have used *in vitro* systems such as pyramidal neurons in acute slices of the mouse neocortex or cultured cerebellar Purkinje cells of the mouse (Jontes and Smith, 2000; Portera-Cailliau et al., 2003; Fujishima et al., 2012). *In vivo* studies mostly used optic tectal neurons of *Xenopus* tadpole and zebrafish larvae, whose brains are transparent, and focused primarily on how dendrites arborize (Wu and Cline, 1998; Rajan et al., 1999; Wu et al., 1999; Sin et al., 2002; Niell et al., 2004; Sanchez et al., 2006; Nagel et al., 2015). The optic tectal neuron has a single dendrite. In contrast, SS neurons, the major type of L4 glutamatergic neurons (65–80% of the total; Fox, 2008), in the mouse barrel cortex have multiple basal dendrites that exhibit an asymmetric pattern strongly oriented toward the barrel center in adulthood, which is the basis of the precise one-to-one relationship between individual whisker stimulation and barrel activation (Lübke et al., 2000; Staiger et al., 2004). Therefore, it is particularly important to determine how the asymmetric dendritic patterns of SS neurons are generated.

It is generally assumed that SS neuron dendrites show symmetric patterns in early neonatal stages and are refined to acquire asymmetric patterns by simply eliminating outer dendrites and adding new inner dendrites and/or elaborating existing inner dendrites during later neonatal stages (Greenough and Chang, 1988; Espinosa et al., 2009; Emoto, 2011; Iwasato, 2020). We have challenged this conventional view by developing *in vivo* imaging approaches for the neonatal mouse cortex to analyze dynamic processes of SS neuron dendritic refinement (Iwasato, 2020). As a first step, we previously conducted *in vivo* imaging of L4 neuron dendrites starting at P5 (9 h interval for 18 h; Mizuno et al., 2014), which provides the first observation of dendritic motility in the mammalian brain *in vivo*. This study revealed that branches of inner dendrites are not only elongated but also often retracted. Similarly, branches of outer dendrites show both elongation and retraction. Our recent longitudinal *in vivo* imaging of L4 neurons in the mouse barrel cortex revealed that SS neurons establish basal dendritic orientation bias through two phases (Nakazawa et al., 2018). During Phase I (by P3), SS neurons produce a larger number of inner basal dendritic trees than outer trees, although both inner and outer trees are similarly primitive in morphology at P3. During Phase II (between P3 and P6), the ratio of inner to outer trees does not change. But individual dendritic trees show extensive turnover. Both inner and outer dendritic trees often disappear quickly. Meanwhile, only a few trees highly elaborate. These “winner” trees emerged specifically from inner trees, which generates a strong orientation bias of SS neuron basal dendrites. Although that study revealed the long-term dynamics that generate orientation bias, the spatiotemporal resolution was not sufficient to capture short-term dynamics. In that study, to cover the whole refinement process between P3 and P6, we set acquisition intervals of 8 or 24 h. However, the changes in dendrite morphology over 8 h are larger than we initially expected, which hindered the detection of rapid changes over shorter periods. For example, temporal resolutions of these studies were not high enough to quantitatively analyze the frequency of emergence and elimination of dendritic branches.

In the current study, we focused on P4, which is the peak time of Phase II (Nakazawa et al., 2018), and asked whether there were differences in short-term dynamics between inner and outer basal dendrites. On the other hand, at this age, it is often difficult to distinguish SS and SP neurons because many SS neurons still have apical dendrites (Nakazawa et al., 2018). Therefore, we also compared dynamics between inner and outer basal dendrites of high-OBI neurons by assuming that the majority of these neurons is prospective SS neurons (for details, see Results). In either the all-L4-excitatory-neuron population or high-OBI neuron population, both inner and outer trees emerged and were eliminated with no marked difference in frequency (Fig. 6). Similarly, inner and outer branches showed comparable frequencies of emergence and elimination and similar behaviors of elongation and retraction (Fig. 6). Thus, although basal dendritic trees and branches of SS neurons were highly dynamic during Phase II, rapid changes in basal dendritic structure did not directly contribute to the formation of asymmetric basal dendritic patterns of SS neurons. Taking all the data from our current and previous *in vivo* imaging studies, we propose that SS neurons establish highly asymmetric dendritic patterns through extensive trial-and-error emergence, elongation, elimination, and retraction of dendritic trees and branches rather than simple emergence/elongation of inner dendrites and elimination/retraction of outer dendrites.

In the current study, we also found that the basal dendritic trees and tips of high-OBI (>0.600) neurons were less dynamic (i.e., exhibited fewer emergent and transient events over the 8 h imaging period) compared with low-OBI (<0.500) neurons (Fig. 7*A*,*B*). It is likely that the majority of high-OBI neurons are of the SS type while low-OBI neurons may include SP neurons and less mature SS neurons (for details, see Results). Our previous study shows that dendritic dynamics at P5 are increased in the absence of NMDA receptor activity (Mizuno et al., 2014). Because NMDA receptor is a key player in dendritic refinement (Datwani et al., 2002; Espinosa et al., 2009; Mizuno et al., 2014), it is possible that dendritic dynamics are reduced with the progress of dendritic refinement.

A caveat of our short-interval imaging is the possible effects of anesthesia on activity-dependent dendritic refinement. During early postnatal stages, sensory cortices show spontaneous activities characterized by correlated firing that is arranged spatially according to the modality, and this correlated activity is important for neuronal circuit refinement (Nakazawa and Iwasato, 2021). In the neonatal barrel cortex, spontaneous activity shows a barrel-corresponding patchwork-typed spatial pattern, and this activity is blocked by general anesthesia with isoflurane (Mizuno et al., 2018). Patchwork spontaneous activity is detected again in ∼5–7 min after stopping the isoflurane supply and fully recovers within 10 min (Mizuno et al., 2018, their Fig. S1D). Similarly, spontaneous activity in the neonatal mouse visual cortex is recovered within 10 min after stopping isoflurane inhalation (Ackman et al., 2012). In the experiment setting of the current study, we started and stopped isoflurane supply 2 min before the start of each imaging session and 2 min before the end of the imaging session, respectively. After finishing each imaging session (20 min), we confirmed that pups woke up and started to interact with littermates within 5 min. Therefore, pups were anesthetized for nearly 30 min in 1 h between two consecutive imaging sessions. Thus, it is likely that the short-term dendritic dynamics of mouse cortical neurons is even faster than what we observed here.

### Specific dendritic features during development revealed by improved spatiotemporal resolution of *in vivo* imaging

The high-spatiotemporal resolution imaging achieved in the current study also enabled us to examine correlations between morphologic features and dendritic tip dynamics (Fig. 8). We found a positive correlation between dendritic tip thickness and its behavior (Fig. 8*D*). Thick tips, which may have dendritic growth cones (Fig. 9), were more likely to be extending (Fig. 8*A*), and thin tips were more likely to be retracting (Fig. 8*B*). Conversely, extending and retracting tips are more likely to be thick and thin, respectively (Fig. 8*E*). Similar correlations between dendritic tip morphology and dendrite behavior are observed in optic tectal neurons in *Xenopus* tadpoles (Wu and Cline, 1998; Hossain et al., 2012) and cortical pyramidal neurons in mouse acute slices (Portera-Cailliau et al., 2003). Thus, it is possible to assume dendritic tip behavior based on dendritic tip morphology revealed by two-photon *in vivo* imaging or confocal imaging of fixed brain slices.

As mentioned above, before *in vivo* time-lapse imaging was achieved, it was generally believed that SS neurons initially have radially oriented basal dendrites, and during critical period of dendritic refinement these neurons specifically prune the outer dendrites to acquire highly asymmetric dendritic patterns (Emoto, 2011; Mizuno et al., 2014). If so, it could be similar to C4da sensory neurons in *Drosophila*, which prune their dendritic arbors during the early metamorphosis. In this pruning process, dendrites are severed and fragmented, and fragments are finally cleared by engulfment (Kuo et al., 2005; Williams and Truman, 2005; Williams et al., 2006; Kanamori et al., 2013). It was wondered whether similar mechanisms may be involved in SS neuron dendritic refinement. By taking advantage of high spatiotemporal resolution of the current imaging, we here carefully observed loci where dendritic segments disappeared within the last 1 h (h7 to h8) combined with *post hoc* confocal imaging of the same loci with high magnification. However, we found no evidence of dendrite severing and fragmentation. This result suggests that the outer basal dendritic trees are unlikely to be pruned from SS neurons in a process akin to that observed for *Drosophila* C4da neurons. In accord with this finding, our previous longitudinal imaging study reveals that SS neurons establish a highly oriented basal dendritic pattern between P3 and P6 without selective elimination of outer dendritic trees. In fact, the numbers of both inner and outer basal dendrites do not differ substantially between P3 and P6 (Nakazawa et al., 2018). Rather, SS neurons establish unique basal dendritic patterns highly oriented toward the barrel center through selection and elaboration of a few inner dendritic trees as winners.

In the current study, we focused our analyses on the short-term dynamics of barrel cortex L4 neurons at P4, the time of peak refinement. Taking our current results and previous longitudinal imaging together, we have revealed highly dynamic features of basal dendrites in the early postnatal period. Rapid dendritic dynamics do not directly contribute to the highly asymmetric basal dendritic pattern of L4 neurons. Instead, dendritic refinement progresses through massive trial-and-error emergence, elongation, elimination, and retraction of dendritic tips and trees.

## Synthesis

Reviewing Editor: Matthew Grubb, King’s College London Decisions are customarily a result of the Reviewing Editor and the peer reviewers coming together and discussing their recommendations until a consensus is reached. When revisions are invited, a fact-based synthesis statement explaining their decision and outlining what is needed to prepare a revision will be listed below. The following reviewer(s) agreed to reveal their identity: Takeshi Imai. Note: If this manuscript was transferred from JNeurosci and a decision was made to accept the manuscript without peer review, a brief statement to this effect will instead be what is listed below.

Review Synthesis:

Both Reviewers are positive about the value and quality of the study. They suggest many alterations that will improve the paper further, so please address each of these as listed below. Please note that after consultation we all agreed that no further experiments are necessary to address any of these comments; rather, additional discussion and/or analyses only are required.

Reviewer 1:

Activity-dependent dendrite patterning is important for the establishment of receptive fields in sensory circuits. The authors pioneered in vivo imaging of this process in neonatal mice (Mizuno et al., 2014). However, the temporal resolution in the previous study was not high enough. In this study, the authors tried in vivo imaging of the barrel cortex at P4 at one-hour intervals. The authors found that dendritic extension and retraction were active in both the inner and outer parts of the barrel. The authors also found that thick tips tended to extend, while thin tips tended to retract.

Overall, the quality of the data is high. This study would be publishable after a major revision, given the scope of the journal. However, there are some issues regarding the interpretation of the data. The writing and presentation also need to be improved, as it is currently difficult to follow the main conclusion of the experiments.

Specific comments:

1) The authors conclude that there is no difference between dendritic dynamics inside and outside the barrel. On the other hand, a strong bias will be established in the end. I, therefore, believe that the authors have overlooked something. The authors should consider some potential problems described below.

1-1) A major concern is the effect of anesthesia. The current model argues that the dendritic patterns are formed based on the barrel-specific “patchwork-type” spontaneous activity. However, spontaneous activity is absent under anesthesia. In this study, the authors used isoflurane anesthesia during the imaging (20 min) and every 1 hour. It is important to check whether the normal spontaneous activity is generated during the interval (40 min). In any case, the activity-dependent events in this experiment should be delayed, and this should be mentioned as a caveat in the discussion.

1-2) According to Mizuno et al. (2014), OBI is already apparent for SS at P4, and it only gradually increased during P4-6. This means that bias in dendritic dynamics during the 8-hour experiment would be very small. The authors may need to obtain more samples if they are to detect such a small difference. Also, the SP data may have masked a potential difference for SS (9/19 neurons). At minimum, the authors need to discuss these issues.

1-3) The length, rather than the number, of extensions/retractions, may account for the bias. This is not investigated in the high-OBI population.

2) The title and abstract describe what was done, but what was found is unclear. The authors need to tell what kind of dynamics and features were newly found. It is also unclear what “trial-and-error (lines 61 and 430)” means here. It should also be noted that the “directed growth (line 62)” model has already been ruled out by Mizuno et al. (2014).

Minor comments:

3) The authors describe “dendrite projection”, but “projection” is inappropriate for dendrites, as they “receive” the inputs. “Dendrite patterning” would be more appropriate.

4) Line 45-46. This sentence is weird. I would rewrite it as follows:

Individual neurons in the sensory cortex establish specific receptive fields based on their dendritic patterns.

5) Line 60. “comparably”

It is unclear what was compared here.

6) Line 69. In vivo imaging is helpful but not essential for studying mechanisms.

7) Line 94. I would cite Ruthazer et al. (Science, 2003).

8) Line 106. Please explain what can be newly studied with in vivo imaging at one-hour intervals.

9) Line 199. Typo. Lecia should be Leica.

10) Line 447. What is known for C4da is not retraction but degeneration. In general, “pruning” refers to both retraction and degeneration (e.g., Luo & O’Leary, 2005). I would rephrase it as follows:

In Drosophila larvae, dendritic pruning of C4da sensory neurons is mediated by degeneration, ∼.

11) Lines 457-459. The results did not support the degeneration model, indeed. But it is not the “evidence against the possibility”. For example, debris may be cleared very quickly. It is also possible that a “nibbling” type of degeneration is involved.

12) Figure 1. “Regular RFP” should be reworded as “cytosolic RFP”. What does “126x” means? Scale bars, rather than magnifications, should be used. “Post-hoc confocal” should be labeled in E.

13) In Figures 2 and 7, different images should be separated by spaces or white lines. In Figures 2, 3, 4, 7-9, the layout should be improved: the panels should be evenly spaced at the same height.

14) Figure 4B. Green/blue and magenta/red are difficult to distinguish. Stable ones should be black for both IN and OUT.

15) I suggest that the authors archive all of the raw images and tracing data in a public repository for future modeling and theoretical studies.

Reviewer 2:

Wang et al. used a high spatiotemporal resolution in vivo imaging approach to characterise the neonatal dendritic refinement of spiny stellate neurons in the mouse barrel cortex. Previous studies with a similar aim were based on image acquisition within long intervals, potentially missing rapid changes in dendritic morphology. Here, hourly time-lapse imaging captured such rapid changes, offering a complete and more comprehensive picture of the postnatal dendritic refinement of SS neurons.

A very positive point about this study is that the authors know that their approach targets both spiny stellate and star pyramid neurons (the former being the majority of the labelled cells), and they explored in depth the dendritic refinement parameters considering both populations.

I strongly recommend this article for publication.

Major comments

I have no major comments.

Minor comments

- Methods section, line 198 until the end of the paragraph: please, specify if these images were stacks and, if so, their dimensions (e.g., total thickness, the step size, and the slides). Information about pixel size should be provided as well.

- Methods section, line 260. Is ‘standard error (SE)’ referring to ‘standard error of the mean (SEM)’? If so, please specify.

- Figure 3: please specify in the figure legend if the example images belong to the same neuron or to different neurons.

- Figure 1E: please, specify ‘confocal’ in the image like in Figure 9B. If ‘126x’ refers to the objective, it should be specified in the figure legend, in both cases.

- Figure legend, figure 2A: ‘P’ should be clarified as ‘postnatal day’ in the same way that ‘E’ was specified as ‘embryonic day’.

- Figure 4: please, find an alternative colour code for ‘OUT’ (magenta) and ‘emerged OUT’ (orange). It is challenging to distinguish both, especially in tree #7.

- Where applicable, whisker-plots should be replaced by dot plots or violin plots (like in Figure 6H). These convey the sample distribution and variability in a more visual way. In that case, the SEM should remain specified in the results section accordingly.

- Table 1, line b): please, specify the distance units.

- Table 1: please, specify here what do the blank spaces mean.

- Methods section, statistical analysis: please, specify if the authors have performed normality tests on the data to determine the appropriate statistical test.

- Line 531: please, remove ‘the’ before ‘the mice’. Alternatively, replace by ‘mice acute slices’.

## Author Response

Response to the Reviewers:

Both Reviewers are positive about the value and quality of the study. They suggest many alterations that will improve the paper further, so please address each of these as listed below. Please note that after consultation we all agreed that no further experiments are necessary to address any of these comments; rather, additional discussion and/or analyses only are required. We were pleased by the positive responses of both reviewers and the editor to our manuscript. We thank both reviewers for their insightful and constructive comments/suggestions, which were extremely helpful for us to improve our manuscript. We have marked all the changes in the manuscript by blue letters. We have addressed all of the reviewers’ concerns as detailed bellow:

(Reviewer 1)

Activity-dependent dendrite patterning is important for the establishment of receptive fields in sensory circuits. The authors pioneered in vivo imaging of this process in neonatal mice (Mizuno et al., 2014). However, the temporal resolution in the previous study was not high enough. In this study, the authors tried in vivo imaging of the barrel cortex at P4 at one -hour intervals.

The authors found that dendritic extension and retraction were active in both the inner and outer parts of the barrel. The authors also found that thick tips tended to extend, while thin tips tended to retract.

Overall, the quality of the data is high. This study would be publishable after a major revision, given the scope of the journal. However, there are some issues regarding the interpretation of the data. The writing and presentation also need to be improved, as it is currently difficult to follow the main conclusion of the experiments.

(ANSWER)

We aim to understand how the asymmetric dendritic pattern of SS neurons are generated during neonatal development. A main conclusion in the current study is “SS neurons acquire biased dendrite patterns through rapid trial-and-error emergence, elongation, elimination, and retraction of dendritic branches and trees.”

It is generally believed that SS neuron dendrites show symmetric patterns in early neonatal stages and are refined to acquire asymmetric patterns by simply eliminating outer dendrites and adding new inner dendrites and/or elaborating existing inner dendrites during later neonatal stages.

We have challenged this conventional view by developing in vivo imaging approaches for the neonatal mouse cortex to analyze dynamic processes of SS neuron dendritic refinement. In the current study, we have conducted short-interval imaging (1-h interval for 8 h). In this study, we were able to capture precise changes in dendrite morphologies. In particular, both barrel-inner and barrel-outer dendrites (trees and branches) emerged/elongated and disappeared/retracted at similarly high frequencies.

As a first step of in vivo imaging of the neonatal mouse cortex, we previously conducted in vivo imaging of L4 neuron dendrites starting at P5 (9-h interval for 18 h) (Mizuno et al., 2014). We subsequently succeeded in longitudinal in vivo imaging from P3 to P6 (8-h interval between P3 to P5 and 24-h interval between P5 to P6). These two studies have contri buted to our understanding of the dynamic mechanisms of dendritic refinement. They revealed that “SS neurons acquire biased dendrite patterns through trial-and-error elongation and retraction of dendritic branches (Mizuno et al., 2014) and trial-and-error emergence and elimination of dendritic trees (Nakazawa et al., 2018).” However, we were not able to analyze emergence and elimination of dendritic branches in previous imaging studies because their spatial resolutions were not high enough. Also, some rapid changes were overlooked in previous studies.

To make these points clearer, we have revised title, abstract, discussion etc accordingly (See below for details) Specific comments:

1) The authors conclude that there is no difference between dendritic dynamics inside and outside the barrel. On the other hand, a strong bias will be established in the end. I, therefore, believe that the authors have overlooked something. The authors should consider some potential problems described below. 1-1) A major concern is the effect of anesthesia. The current model argues that the dendritic patterns are formed based on the barrel-specific “patchwork-type” spontaneous activity. However, spontaneous activity is absent under anesthesia. In this study, the authors used isoflurane anesthesia during the imaging (20 min) and every 1 hour. It is importa nt to check whether the normal spontaneous activity is generated during the interval (40 min). In any case, the activity -dependent events in this experiment should be delayed, and this should be mentioned as a caveat in the discussion.

(ANSWER)

Thank you for pointing a very important issue. As this reviewer mentioned, patchwork-type spontaneous activity in the neonatal mouse barrel cortex layer 4 is absent under the general anesthesia by isoflurane (Mizuno et al., 2018). Spontaneous activity is detected again in 5∼7 minutes after stopping the isoflurane supply and fully recovers within 10 minutes (See Fig. S1D of Mizuno et al., 2018 attached below). Similarly, spontaneous activity in the neonatal mouse visual cortex is recovered within 10 minutes after stopping isoflurane inhalation (Ackman et al., 2012). (Figure S1D, Mizuno et al., 2018)

In the experiment setting of the current study, we started and stopped isoflurane supply 2 minutes before the start of the imaging session and 2 minutes before the end of the imaging session, respectively. After finishing each imaging session, we confirmed that pups woke up and started to interact with littermates within 5 minutes.

Therefore, cortical spontaneous activity should be present for about 30 minutes in 1 hour. We agree with th e reviewer that activity -dependent events could be delayed in our imaging condition. We have mentioned this issue as a caveat in Discussion (p.23). 1-2) According to Mizuno et al. (2014), OBI is already apparent for SS at P4, and it only gradually increased during P4 -6. This means that bias in dendritic dynamics during the 8-hour experiment would be very small. The authors may need to obtain more samples if they are to detect such a small difference. Also, the SP data may have masked a potential difference for SS (9/19 neurons). At minimum, the authors need to discuss these issues.

(ANSWER)

(1) OBI is only an index of orientation bias. Our recent longitudinal in vivo imaging study of L4 neurons in the mouse barrel cortex (Nakazawa et al., 2018) revealed that SS neuron dendrites already show orientation bias at P3 but this orientation bias and orientation bias at P6 are qualitatively and quantitatively different. At P3, SS neurons have a larger number of inner basal dendritic trees than outer trees, although both inner and outer trees are similarly primitive in morphology. Then between P3 and P6 (Phase II), the ratio of inner to outer trees does not chan ge. Meanwhile, only a few trees highly elaborate. These “winner” trees emerged specifically from inner trees, which generates a strong orientation bias of SS neuron basal dendrites. Thus, Phase II (between P3 and P6) is an important period for dendritic re finement, and P4 is the middle of Phase II. This is why we focused on P4 in the current study.

(2) As this reviewer suggest s and also judging from results of our previous studies (Mizuno et al., 2014; Nakazawa et al., Nature Commun. 2018), we believe that if we collect a huge number of samples we should be able to detect significant differences between inner and outer dendrites in some aspects of dendrite behavior. But the aim of the current study was not necessarily to detect significant differences in such small differences. Instead, we wanted to know precise dendritic behavior during the middle of Phase II of dendritic refinement. Therefore, small differences between inner and outer dendrites are themselves important information.

(3) In the current study, we chose P4, which is the middle of Phase II . At this age, majority of SS neurons still have apical dendrite and therefore it was difficult to distinguish SS and SP cells precisely (Nakazawa et al., 2018). However, according to the Nakazawa et al., 2018 paper ’s data, which retrospectively identified prospective SS neurons even before the initiation of apical dendrite retraction, we can assume that high OBI neurons at P4 are most likely prospective SS neurons. See Results (Lines 380-404)

To make these points clearer, we have revised the Discussion extensively (Lines 492-533). Also, we have revised Abstract accordingly. 1-3) The length, rather than the number, of extensions/retractions, may account for the bias. This is not investigated in the high-OBI population.

(ANSWER)

The length of extensions/retractions were similar between high vs low-OBI (Fig. A as below), high-OBI IN vs high-OBI OUT (Fig. 6H), and low-OBI IN vs low-OBI OUT (Fig. B as below) groups. Fig. A Fig. B

Again, if we collect a huge number of samples, inner dendrites should show larger extension and larger stability than outer dendrites. But the aim of the current research is not necessarily to detect statistical significance in such small differences. In the current study, we were most interested in quantification of emergence and elimination of dendritic branches because they were difficult to be analyzed in previous long -interval imaging. Only in short-interval imaging in the current study, we were able to characterize these dynamics.

2) The title and abstract describe w hat was done, but what was found is unclear. The authors need to tell what kind of dynamics and features were newly found. It is also unclear what “trial-and-error (lines 61 and 430)” means here.

(ANSWER)

We have revised the title and abstract, accordingly. What was found in the current study is as follows: “SS neurons acquire biased dendrite patterns through rapid trial-and-error emergence, elongation, elimination, and retraction of dendritic branches and trees .”. In particular, quantification of emergence and elimination of dendritic branches is important, because these analyses were difficult to be done in previous imaging studies with 8 -9 h High OBI Low OBI -40 -20 0 20 40 60 Length changes in 1 hour (μm) ns IN OUT -40 -20 0 20 40 60 Low OBI neurons Length changes in 1 hour (μm) ns imaging intervals. To clarify the meaning of “trial-and-error” here, we have revised Abstract (Lines 60-61) and Results (Lines 432-433). It should also be noted that the “directed growth (line 62)” model has already been ruled out by Mizuno et al. (2014).

(ANSWER)

I assume that you know our “Dynamic model” of dendritic refinement mechanisms, which was proposed for the first time in the Mizuno et al., 2014 paper. But unfortunately, we feel that our model is not widely accepted yet.

Many researchers still use so called the “Directed growth model” (“Steady growth model”). Therefore, we need to repeatedly refine and propose our model with new lines of evidence. In the current study, we have provided new evidence with high spatiotemporal resolution imaging.

However, probably it is too much for Abstract. Therefore, we have deleted this passage from Abstract and explained it in Discussion (Lines 492- 533).

Minor comments:

3) The authors describe “dendrite projection”, but “projection” is inappropriate for dendrites, as they “receive” the inputs. “Dendrite patterning” would be more appropriate.

(ANSWER)

We have changed “dendrite projection” to “dendrite patterning” accordingly .

4) Line 45-46. This sentence is weird. I would rewrite it as follows:

Individual neurons in the sensory cortex establish specific receptive fields based on their dendritic patterns.

(ANSWER)

We have revised the sentence as suggested. Thank you so much.

5) Line 60. “comparably” It is unclear what was compared here.

(ANSWER)

We have changed “comparably” to “similarly”.

6) Line 69. In vivo imaging is helpful but not essential for studying

mechanisms.

(ANSWER)

We agree with the reviewer. We have changed “required” to “useful”.

7) Line 94. I would cite Ruthazer et al. (Science, 2003).

(ANSWER)

Added.

8) Line 106. Please explain what can be newly studied with in vivo imaging at one-hour intervals.

(ANSWER)

Revised. Also see my answers above.

9) Line 199. Typo. Lecia should be Leica.

(ANSWER)

Corrected. Thank you so much.

10) Line 447. What is known for C4da is not retraction but degeneration. In general, “pruning” refers to both retraction and degeneration (e.g., Luo & O’Leary, 2005). I would rephrase it as follows: In Drosophila larvae, dendritic pruning of C4da sensory neuro ns is mediated by degeneration, ∼.

(ANSWER)

Revised. Thank you so much.

11) Lines 457-459. The results did not support the degeneration model, indeed. But it is not the “evidence against the possibility”. For example, debris may be cleared very quickly. It is also possible that a “nibbling” type of degeneration is involved.

(ANSWER)

Thank you so much for the useful comment. We agree with the reviewer. We have revised Results (Lines 460-461) and Discussion (Lines 573-592), accordingly.

12) Figure 1. “Regular RFP” should be reworded as “cytosolic RFP”. What does “126x” means? Scale bars, rather than magnifications, should be used. “Post-hoc confocal” should be labeled in E.

(ANSWER)

We have revised Figure 1, Fig. 1 legend, Results and Methods, accordingly.

13) In Figures 2 and 7, different images should be separated by spaces or white lines.

(ANSWER)

Revised accordingly.

In Figures 2, 3, 4, 7-9, the layout should be improved: the panels should be evenly spaced at the same height.

(ANSWER)

Improved.

14) Figure 4B. Green/blue and magenta/red are difficult to distinguish. Stable ones should be black for both IN and OUT.

(ANSWER)

We now use cyan and magenta arrowheads to mark newly emerged inner and outer branches (and trees), respectively.

15) I suggest that the authors archive all of the raw images and tracing data in a public repository for future modeling and theoretical studies.

(ANSWER)

We will consider to archive our raw images and tracing data in a public repository. Can you suggest appropriate public repositories for these data?

(Reviewer 2)

Wang et al. used a high spatiotemporal resolution in vivo imaging approach to characterise the neonatal dendritic refinement of spiny stellate neurons in the mouse barrel cortex. Previous studies with a similar aim were based on image acquisition within long intervals, potentially missing rapid changes in dendritic morphology. Here, hourly time -lapse imaging captured such rapid changes, offering a complete and more comprehensive picture of the postnatal dendritic refinement of SS neurons.

A very positive point about this study is that the authors know that their approach targets both spiny stellate and star pyramid neurons (the former being the majority of the labelled cells), and they explored in depth the dendritic refinement par ameters considering both populations. I strongly recommend this article for publication.

Major comments

I have no major comments.

Minor comments

- Methods section, line 198 until the end of the paragraph: please, specify if these images were stacks and, if so, their dimensions (e.g., total thickness, the step size, and the slides). Information about pixel size should be provided as well.

(ANSWER)

We have revised the Methods section (P.9) and figure legends.

- Methods section, line 260. Is ‘standard error (SE)’ referring to ‘standard error of the mean (SEM)’? If so, please specify.

(ANSWER) Revised.

- Figure 3: please specify in the figure legend if the example images belong to the same neuron or to different neurons.

(ANSWER)

A-C are images from the same neuron (neuron #1 in Table 1). D is from neuron #3 in Table 1. We have added this information in the figure legend.

- Figure 1E: please, specify ‘confocal’ in the image like in Figure 9B. If ‘126x’ refers to the objective, it should b e specified in the figure legend, in both cases.

(ANSWER)

We have revised Fig.1E and its legend.

- Figure legend, figure 2A: ‘P’ should be clarified as ‘postnatal day’ in the same way that ‘E’ was specified as ‘embryonic day’.

(ANSWER) Revised.

- Figure 4: please, find an alternative colour code for ‘OUT’ (magenta) and ‘emerged OUT’ (orange). It is challenging to distinguish both, especially in tree #7.

(ANSWER)

We now use cyan and magenta arrowheads to mark newly emerged inner and outer branches (and trees), respectively.

- Where applicable, whisker-plots should be replaced by dot plots or violin plots (like in Figure 6H). These convey the sample distribution and variability in a more visual way. In that case, the SEM should remain specified in the results section accordingly.

(ANSWER)

We have revised Figs. 5B, 5E, 5H, 7A, 7B, 8E, accordingly.

- Table 1, line b): please, specify the distance units.

(ANSWER) Revised.

- Table 1: please, specify here what do the blank spaces mean.

(ANSWER) Revised.

- Methods section, statistical analysis: please, specify if the authors have performed normality tests on the data to determine the appropriate statistical test.

(ANSWER)

We have performed normality tests for most data. But we eventually chose non-parametric tests for all statistical analyses.

- Line 531: please, remove ‘the’ before ’the mice’. Alternatively, replace by ‘mice acute slices’.

(ANSWER) Revised
